# A New Twist in Protein Kinase B/Akt Signaling: Role of Altered Cancer Cell Metabolism in Akt-Mediated Therapy Resistance

**DOI:** 10.3390/ijms21228563

**Published:** 2020-11-13

**Authors:** Isabell Götting, Verena Jendrossek, Johann Matschke

**Affiliations:** Institute of Cell Biology (Cancer Research), University Hospital Essen, University of Duisburg-Essen, 45147 Essen, Germany; isabell.goetting@uk-essen.de (I.G.); verena.jendrossek@uni-due.de (V.J.)

**Keywords:** Akt, metabolism, DNA repair, DDR, metabolites, protein modifications, chromatin modifications, energy metabolism, antioxidant defense

## Abstract

Cancer resistance to chemotherapy, radiotherapy and molecular-targeted agents is a major obstacle to successful cancer therapy. Herein, aberrant activation of the phosphatidyl-inositol-3-kinase (PI3K)/protein kinase B (Akt) pathway is one of the most frequently deregulated pathways in cancer cells and has been associated with multiple aspects of therapy resistance. These include, for example, survival under stress conditions, apoptosis resistance, activation of the cellular response to DNA damage and repair of radiation-induced or chemotherapy-induced DNA damage, particularly DNA double strand breaks (DSB). One further important, yet not much investigated aspect of Akt-dependent signaling is the regulation of cell metabolism. In fact, many Akt target proteins are part of or involved in the regulation of metabolic pathways. Furthermore, recent studies revealed the importance of certain metabolites for protection against therapy-induced cell stress and the repair of therapy-induced DNA damage. Thus far, the likely interaction between deregulated activation of Akt, altered cancer metabolism and therapy resistance is not yet well understood. The present review describes the documented interactions between Akt, its target proteins and cancer cell metabolism, focusing on antioxidant defense and DSB repair. Furthermore, the review highlights potential connections between deregulated Akt, cancer cell metabolism and therapy resistance of cancer cells through altered DSB repair and discusses potential resulting therapeutic implications.

## 1. Introduction

Radiotherapy, in addition to surgery and chemotherapy, is one of the three main standard therapies for cancer treatment. The lethal effects of ionizing radiation (IR) on cancer cells are based on the radiation-induced damage to cellular macromolecules [1,2,3,4,5]. Herein, the induction of double-strand breaks (DSBs) is considered as the most lethal type of radiation-induced cell damage [1,2,3,4,5]. Therefore, it is not surprising that cells have evolved a complex DNA damage response (DDR) in order to protect their DNA from lethal DNA DSBs [6]. As part of the DNA damage response, DNA DSB can be repaired via two main mechanisms: (i) the high-fidelity, but cell cycle-dependent homologous recombination repair (HRR) pathway, and (ii) the cell cycle-independent but error-prone classical non-homologous end joining (NHEJ) repair pathway, though further alternative pathways exist (for a review see [2,4,7,8,9]). Proper DSB repair through the various pathways depends among others on the recognition of the DNA damage and on the presence of a set of specific repair proteins. Moreover, appropriate DNA damage signaling and DSB repair also depend on histone and DNA modifications as well as on chromatin remodeling [10,11,12,13,14,15,16]. Furthermore, beyond direct induction of DNA damage, exposure to ionizing radiation induces the formation of reactive oxygen species (ROS), which oxidize cellular macromolecules, e.g., lipids, protein, RNA and DNA, and can indirectly contribute to the formation of single strand breaks (SSB) and DSBs [17,18]. Consequently, cells have also evolved a cellular response to ROS to counteract ROS-mediated damage cellular by activation of antioxidant systems [17,18]. Importantly, recent reports support a requirement of metabolic activity and certain metabolites to fuel the DDR, as well as antioxidant defense and DSB repair [19,20,21,22].

Of note, the ability of cancer cells to mount an efficient DDR and the high capacity of cancer cells to repair radiation-induced lethal DNA lesions promotes radioresistance, and this can be exploited therapeutically [1,23]. Increased basal activation of the DDR or increased antioxidant capacity can also contribute to increased radiation resistance [22,24,25]. In fact, DDR, DSB repair and cellular antioxidant defense systems are recognized as important targets for improving the outcome of cancer (radio)therapy [9,24,26,27,28,29,30]. However, oncogene-induced and stress-induced metabolic reprogramming emerged as an additional important hallmark of cancer [31]. Notably, the resulting metabolic plasticity affects the primary cancer response to therapy and also supports adaptive therapy resistance, including treatments involving radiotherapy [22,24,32,33]. Vice versa, the frequent occurrence of defects in DDR and DSB repair pathways in various cancers offers opportunities for synthetic lethality approaches by inhibition of complementary DSB repair pathways [34,35,36,37,38]. Excitingly, factors beyond genetic defects in core proteins of DDR and DSB repair can also promote DSB repair defects: these include for example deregulated expression, mutations in chromatin modifiers or metabolic enzymes [39,40,41,42] as well as tumor hypoxia [43,44,45].

Until now, many studies have investigated the areas of DNA damage response and cancer metabolism separately. However, the above observations highlight the importance to connect these two research areas for providing a scientific basis to optimize concepts for rational combinatorial treatments. For example, the phosphatidylinositol-3-kinase (PI3K)-Akt pathway is frequently deregulated in cancer [46,47,48,49]. Aberrant activation of this pathway supports cancer development and progression as well as resistance to standard or molecularly targeted cancer therapies through a variety of cellular activities. These include for example protein synthesis, cell cycle progression, proliferation, survival, angiogenesis, metastasis and cancer metabolism [46,50]. However, the survival kinase Akt also exerts multifaceted roles in regulating the DDR and the repair of radiation-induced or chemotherapy-induced DSB [46,47,48,49] (for a review see [50,51,52]). Even more important, isoform-dependent or activation-associated activities of one of the three Akt isoforms Akt1, 2 or 3 have been associated with improved DNA repair and radiation resistance [53,54,55,56,57,58].

**Figure 1:**
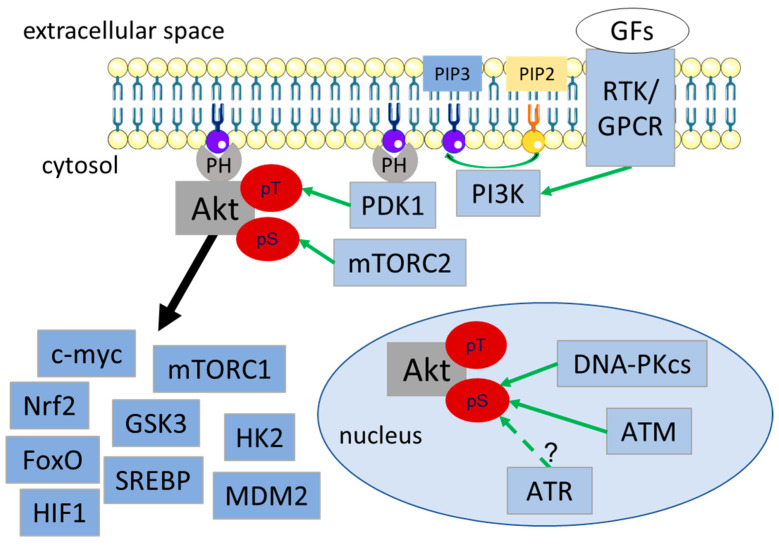
Schematic representation of compartmentalized Akt activation via receptor tyrosine kinases and intracellular regulators and important Akt target proteins. Regulators of Akt are indicated in light blue and exemplary Akt target proteins in dark blue. Activation is shown with green arrows. Black arrow indicates direct or indirect regulation/phosphorylation. Abbreviations: ataxia telangiectasia mutated (ATM), ataxia telangiectasia and Rad3 related (ATR), DNA-dependent protein kinase, catalytic subunit (DNA-PKcs), Forkhead-Box-Protein O3 (FoxO), growth factors (GFs), G-protein couple receptor (GPCR), glycogen synthase kinase 3 (GSK), Hypoxia inducible factor 1 (HIF1), Hexokinase 2 (HK2), Mouse double minute 2 homolog (MDM2), mechanistic target of rapamycin/mammalian target of rapamycin (mTOR), nuclear factor erythroid 2-related factor 2 (Nrf2), phosphoinositide-dependent kinase 1 (PDK1), pleckstrin homology (PH), Phosphatidyl-inositol-3-kinase (PI3K), Receptor tyrosine kinase (RTK), Sterol regulatory element binding proteins (SREBP).

The biochemical processes responsible for activation of Akt and major physiological targets are well known and will only be summarized briefly here (Figure 1). Activation of PI3K by, e.g., receptor tyrosine kinases (RTKs) or G-protein coupled receptors (GPCRs), leads to the generation of phosphatidylinositol-3,4,5-trisphosphate (PIP3) in the cytoplasmic membrane; this mediates recognition and recruitment of Pleckstrin Homology (PH) domain-containing proteins to PIP3-rich regions in the cytoplasmic membrane, such as Akt, phosphoinositide-dependent kinase 1 (PDK1) or PH-domain containing phosphatases [50,59,60,61,62,63]. Co-localization of PDK1 and Akt at the membrane facilitates PDK1-dependent phosphorylation of Akt at Thr308 [50]. The second activation-associated phosphorylation site at Ser473 undergoes compartment-specific phosphorylation through either mammalian target of rapamycin (mTORC2) (cytosol), integrin linked kinase (focal adhesion), or DNA-dependent protein kinase catalytic subunit (DNA-PKcs), ataxia telangiectasia mutated (ATM) and presumably also ataxia telangiectasia and Rad3 related (ATR) (nucleus), leading to its full activation (Figure 1) [64,65,66,67,68,69,70] (for a detailed review see [50,51]).

Beyond phosphorylation, phosphatase-mediated degradation of PIP3 or deactivation of Akt itself represent important regulatory signaling hubs—on the one hand, by dephosphorylating lipid second messengers such as PI(3,4)P2 and PIP3, the lipid phosphatases Inositol polyphosphate 4-phosphatase type II (INPP4B) and phosphatase and tensin homologue (PTEN) prevent membrane recruitment and activation of Akt by PDK1 [71,72,73]. On the other hand, the protein phosphatases PP2A and PHLPP1/2 dephosphorylate Akt on T308 and S473, respectively, thereby terminating Akt activity [60,61].

Akt acts as a Ser/Thr-specific kinase that either directly regulates cellular processes by phosphorylating various effector proteins or indirectly by phosphorylating key regulators of signaling networks or transcription factors. These include, for example, the transcription factors, such as c-myc, nuclear factor erythroid 2-related factor 2 (Nrf2), hypoxia-inducible factor (HIF1), forkhead box O transcription factors (FoxO) and sterol regulatory element binding proteins (SREBP) (Figure 1) [74]. Furthermore, Akt-dependent phosphorylation inhibits the activity of glycogen synthase kinase 3 (GSK3). Since GSK3β-dependent phosphorylation leads to ubiquitination and degradation of cyclin D1, Mcl-1 or c-myc, Akt-dependent inhibition of GSK3β promotes proliferation and survival [50,75,76,77]. Furthermore, Akt-dependent regulation of apoptosis is mediated by inhibition of p53 through phosphorylation and activation of the p53 inhibitor mouse double minute 2 homolog (MDM2) [50]. Akt also regulates proteins of the FoxO transcription factor family by phosphorylation in the nuclear translocation sequence [50,78,79].

Active Akt also promotes the activation of the protein kinase complex mTORC1 though inhibitory phosphorylation of tuberous sclerosis complex 2 (TSC2). Activation of mTORTC1 leads to activation of anabolic processes, cell growth and inhibition of autophagy; herein, activation of protein, lipid and nucleotide synthesis involves mTORC1-dependent activation of the transcription factor SREBP [50,80,81,82,83,84,85]. mTORC1 functions as a sensor of stress and certain nutrients as it is activated in response to growth factors and high levels of amino acids, oxygen, nutrients and energy [80]. The stress sensor AMPK functions as a counterpart of mTORC1, which senses energy deprivation to promote energy producing processes in the cell [18].

Taken together, the survival kinase Akt exerts multifaceted roles in regulating the DDR, DSB repair and cell survival, particularly under stress conditions, thereby contributing to therapy resistance (for a review see [50,51,52]). In the present review, we will highlight a new facet of Akt-dependent therapy resistance by regulation of metabolic pathways with relevance to the DDR, DSB repair and radiation resistance.

## 2. Role of Akt in DNA Damage Response and DSB Repair

**Figure 2:**
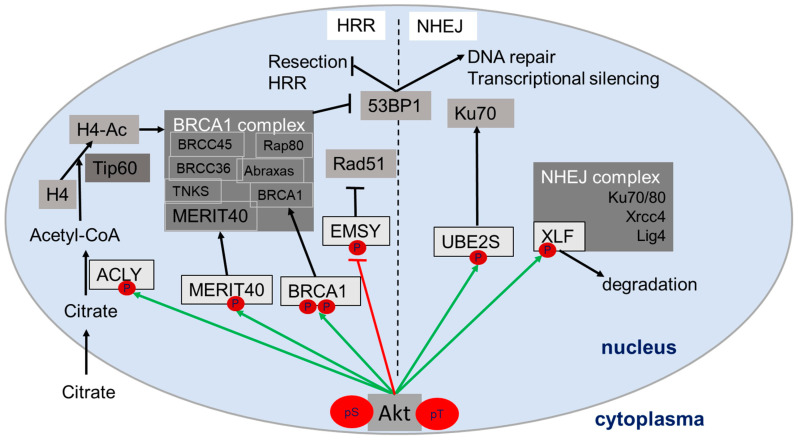
Schema of direct and indirect Akt-dependent regulation of various proteins in the nucleus involved in homologous recombination repair (HRR) or non-homologous end-joining (NHEJ). Akt target proteins are indicated in light grey, Akt-dependent activation/stabilization with green arrows and Akt dependent deactivation/degradation with red block arrow. Black lines indicate subsequent reaction steps in signaling (metabolic reaction, posttranslational modification, degradation). For details about the assumed consequences for the action of the nuclear Akt target proteins see main text paragraph 2. Abbreviations: tumor suppressor p53-binding protein (53BP1); ATP-citrate lyase (ACLY); Breast cancer type 1 susceptibility protein (BRCA1); BRCA1/BRCA2-containing complex (BRCC); BRCA2-interacting transcriptional repressor (EMSY); histone 4 (H4); ligase 4 (lig4); mediator of Rap80 interactions and targeting 40 kDa (MERIT40); receptor-associated protein 80 (Rap80); tankerase (TNKS); Ubiquitin conjugating enzyme E2 S (UBE2S); XRCC4-like factor (XLF); X-ray repair cross-complementing protein 1 (Xrcc4).

Interestingly, research during the last decades linked Akt to the regulation of the DNA damage response and DNA repair (Figure 2). In fact, in response to DNA damage, DNA-PKcs, ATM and presumably also ATR can phosphorylate and activate Akt at S473 in the nucleus leading to its full activation [67,68,69,70]. Once activated, Akt regulates different proteins in DSB repair through NHEJ and HRR, potentially in an isoform-specific manner with suggested impact on DNA repair and the outcome of genotoxic therapies ([54,55,56,57]; for a review see [51]). For instance, Akt-dependent phosphorylation of the XRCC4-like factor (XLF) on Thr181 triggers dissociation of XLF from its complex with DNA ligase IV and XRCC4 [86]. It has been proposed that the release of XLF from this repair complex promotes subsequent XLF degradation [86] or alternatively facilitates DNA repair dynamics [67]. UBE2S is another nuclear target protein of Akt with a suggested role in NHEJ: Akt-dependent phosphorylation of UBE2S at Thr152 protected the protein from proteasomal degradation [87], thereby presumably increasing resistance to DNA damage [88]. Herein, Hu and colleagues suggested a role of UBE2S in regulating NHEJ through binding to Ku70, whereas Paul and colleagues proposed a role of UBE2S in RNF8-mediated transcriptional silencing near DNA damage sites [87], [89]. With respect to HRR, Akt can phosphorylate the breast cancer type 1 susceptibility protein (BRCA1) at Ser694 and Thr509 [90,91], and thereby inhibit BRCA1’s proteasomal degradation [91]. In this way, active Akt prolongs BRCA1-mediated suppression of 53BP1 activity, and thus, favors resection of DNA and HRR while suppressing repair by NHEJ [92]. In addition, Akt phosphorylates a protein of the BRCA1 complex, the mediator of Rap80 interactions and targeting 40 kDa (MERIT40) on S29 [93,94,95]. It has been proposed that Akt-dependent MERIT40 phosphorylation leads to the stabilization of the BRCA1 complex, improved DNA repair via HRR and resistance to doxorubicin [93,94,95]. Finally, Akt-dependent phosphorylation of the BRCA2-interacting transcriptional repressor (EMSY) on T207 inhibits the suppressive action of EMSY on Rad51 foci formation and the resulting impairment of HRR [96,97].

Taken together, Akt-dependent regulation of its nuclear target proteins mostly improves DNA repair and can thereby promote resistance to genotoxic therapies [51,56,88,93,94,95,98]. Furthermore, Akt-dependent regulation of cell survival, growth and proliferation can indirectly influence the radiation response to promote therapy resistance [51,52,99]. Therefore, it is highly attractive to use inhibitors of Akt and its downstream targets in a combination with other treatments to improve treatment outcome (reviewed in [99]).

## 3. Relevance of Metabolic Processes in the DDR

The findings of Otto Warburg revealed, already over more than 50 years ago, that cancer cells consume more glucose than normal tissue, which is a consequence of increased glycolytic activity of the cells [100]. In fact, increased metabolic activity and reprogramming are nowadays considered as important characteristics and emerging hallmarks of cancer cells defined by Hanahan and Weinberg in 2011 [31,101]. High metabolic activity in cancer cells is required for the generation of macromolecules, growth, proliferation and cellular homeostasis [101,102]. Emerging evidence further points to an interplay between cancer metabolism and therapy resistance [101].

As mentioned above, therapy-induced DSB are considered as the most lethal lesions caused for example by exposure to ionizing radiation [1,2,3,4,5]. Therefore, the most important mechanisms causing resistance to radiotherapy and other genotoxic cancer therapies involve improved antioxidant defense to detoxify DNA damaging ROS, intrinsic activation of the DDR, and increased DSB repair capacity [1,27,29,103]. Interestingly, evidence is increasing that cells not only rely on the integrity of the core DSB repair pathways, but also on proper epigenetic signaling (e.g., via posttranslational modification of histones and DNA) and chromatin remodeling for effective DSB repair [10,11,14], [15,104,105]. These processes require the generation of essential metabolites, e.g., phosphate in form of ATP, C1 (methyl) and C2 (acetyl) moieties, ubiquitin, ADP-ribose, nucleotides and glutathione (GSH) [101,106,107]. These observations make the cellular metabolism an important determinant for a successful DDR and DSB repair. In fact, various reports support a requirement of metabolic activity or of certain metabolites to fuel antioxidant defense, DDR or DSB repair [101,106,107].

In the following paragraphs, we will highlight different metabolic pathways with relevance to antioxidant defense, DDR and DSB repair, and thus potential relevance to cell survival upon exposure to ionizing radiation.

### 3.1. Reactive Oxygen Species and Antioxidant Defense

The presence of ROS leads to the activation of transcription of genes involved in the antioxidant defense; these are mainly regulated by the stress sensors AMPK and Akt via the transcription factor Nrf2 but also in part via FoxO and HIF1, respectively [18,108]. FoxO, Nrf2 or HIF1-mediated transcription leads to the expression of various antioxidant enzymes: these include ROS-detoxifying enzymes such as superoxide dismutase (SOD) and catalase (CAT), or components of the thioredoxin (TrX) or glutathione-glutaredoxin (GrX) antioxidant systems such as nicotinamide adenine dinucleotide phosphate (NADPH), thioredoxin reductase, thioredoxin, glutathione reductase, reduced glutathione (GSH) and Grx [109,110]. The molecular function of the antioxidant systems is the generation of reductive equivalents and mostly involves thiol-dependent peroxidases, which regulate the dithiol/bisulfide balance and counteract toxic ROS [109,110]. Cells with higher levels of antioxidants such as NADPH and GSH have a higher capacity to reduce therapy-induced ROS and by that can be more radioresistant [17,24,26,33,109,111,112]. Beyond reducing levels of toxic ROS, cellular antioxidants can also reactivate fatty acid oxidation (FAO) and increase ATP levels in cancer cells, and thereby further supporting cell survival under therapy [113]. It has been demonstrated that limiting provision or regeneration of cellular antioxidants is suited to counteract increased radioresistance of cancer cells with high antioxidant capacity [17,24,26,33,109,111,112]. Overall, inhibition of pathways responsible for antioxidant provision, regeneration or function represents an attractive strategy to increase the cytotoxicity of ROS-inducing treatments including radiotherapy [17,24,33,103,112,114,115].

### 3.2. Nucleotide Synthesis

**Figure 3:**
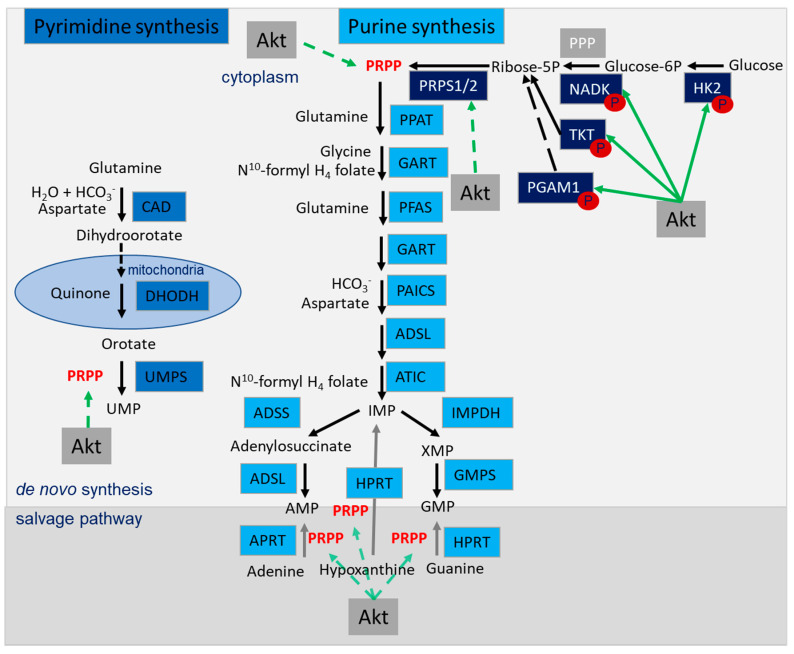
Nucleotide synthesis pathway: de novo synthesis of pyrimidines and purines and salvage pathway, utilizing phospho-ribose pyrophosphate (PRPP) produced in Akt-regulated pathways to generate uridine monophosphate (UMP), adenosine monophosphate (AMP) and guanosine monophosphate (GMP). Enzymes of the pyrimidine synthesis in light blue, enzymes of the purine synthesis in turquoise, enzymes involved in generating PRPP in dark blue. Akt-dependent activation is marked with green arrows, the metabolic pathway of de novo synthesis is marked with black arrows and the salvage pathway is marked with grey arrows. Abbreviations: pyrimidine synthesis: carbamoyl-phosphate synthetase (CAD), dihydroorotase, dihydroorotate dehydrogenase (DHODH), uridine monophosphate synthetase (UMPS); purine synthesis: adenylosuccinate lyase (ADSL), 5-aminoimidazole-4-carboxamide ribonucleotide formyltransferase (ATIC), glycinamide ribonucleotide transformylase (GART), phosphoribosylaminoimidazole carboxylase and phosphoribosylamino-imidazolesuccinocarboxamide synthase (PAICS), phosphoribosyl-formylglycinamidine synthase (PFAS), phosphoribosyl pyrophosphate amidotransferase (PPAT); the salvage pathway: adenylosuccinate synthase (ADSS), adenine phosphoribosyltransferase (APRT) guanine monophosphate synthase (GMPS), hypoxanthine phosphoribosyltransferase (HPRT), inosine monophosphate dehydrogenase (IMPDH); generation of PRPP; hexokinase 2 (HK2), NAD kinase (NADK), phosphoglycerate mutase 1 (PGAM1), pentose phosphate pathway (PPP), ribose-phosphate pyrophosphokinase 1/2 (PRPS1/2), transketolase (TKT).

Nucleotides are essential for cell proliferation (DNA replication) but also for DNA repair processes, especially for HRR that involves novel synthesis of DNA [20,116]. Nucleotide provision depends on the generation of purines and pyrimidines which are either synthesized de novo; alternatively, purines and pyrimidines can be generated in the salvage pathway, which is among others fueled by the folate pathway (Figure 3) [117]. De novo synthesis of pyrimidines requires different enzymes starting with carbamoyl-phosphate synthetase (CAD), dihydroorotase, dihydroorotate dehydrogenase (DHODH) and uridine monophosphate synthetase (UMPS). These enzymes use glutamine, aspartate and phospho-ribose pyrophosphate (PRPP), the active form of ribose-5-phosphate, which is generated in the pentose phosphate pathway (PPP) (Figure 3) [118]. Glutamine, aspartate and PRPP are also needed beyond glycine and folate for generation of IMP during de novo purine synthesis (Figure 3) [118]. Importantly, de novo nucleotide synthesis and salvage pathways as well as their upstream regulatory processes represent valuable targets for anticancer therapy [119]. Even more important, Akt has been identified as an important regulator of both processes: this includes direct regulation of proteins involved in glycolysis and the PPP and indirect regulation through modulation of PRPP-provision (Figure 3; see also Section 4.6).

### 3.3. Basic Metabolites Required for the Modification of Proteins, DNA and Chromatin

Many proteins involved in DSB repair pathways undergo posttranslational modifications such as phosphorylation/dephosphorylation [120]. Posttranslational modifications of DDR proteins such as ATM, ATR, DNA-PKcs, Ku70/80 are important for their function, their recruitment to sites of DNA damage or both [120]. Furthermore, modification of the DNA and of proteins in the chromatin context is another factor influencing the DDR and successful DSB repair with impact on the maintenance of genome and epigenome integrity. In fact, changes in DNA and chromatin structure have been implicated in the activation and transmission of the DDR and DSB repair, while local chromatin condensation has been reported to support DDR signaling and the ligation of broken DNA ends in close proximity, chromatin remodeling or relaxation, depletion and re-assembly of nucleosomes. Additionally, histone modifications are required to make DNA breaks accessible to the repair machinery for proper DSB repair [10,11,12,13,14,15,16,121]. Finally, inhibition of replication and transcriptional silencing at sites of DNA damage by the recruitment of silencing factors, DNA methylation and repressive histone marks helps to avoid deleterious interference between transcription and repair machineries or DNA replication and repair [120,122,123]. In this context, DNA methylation/demethylation and histone modifications such as acetylation/deacetylation, methylation/demethylation and ubiquitination/de-ubiquitination are not only involved in regulation of gene transcription, but also determine the accessibility of the DNA during DSB repair or alternatively serve as assembly platforms for repair proteins when accumulating around sites of DSBs as thoroughly reviewed elsewhere [15,104,124,125].

As this review focuses on metabolic requirements for DSB repair, we will briefly introduce the main metabolites required for the modification of DNA and proteins during the DDR. These include ATP, NAD+, methyl- and acetyl-groups as well as the enzymes catalyzing the respective posttranslational modifications with essential function for proper DDR and DSB repair (for a review see [120,126]). Ubiquitination reactions are also important for transmission of the DDR and for ensuring dynamic access and removal of repair proteins and associated factors at DNA damage sites [127,128,129]. However, ubiquitin is not considered as a limiting metabolite and will, therefore, not be discussed in detail here.

In brief, as the best-known example, ATM, ATR and DNA-PKcs catalyze phosphorylation of H2A.X to recruit core DNA repair factors to sites of DNA damage [13,130,131]. The phosphorylation of H2A.X and of other proteins reaction requires high energy phosphate, e.g., in the form of ATP generated during glycolysis or oxidative phosphorylation [132,133,134,135].

Acetyl-CoA is the main acetyl-group donor for acetylation-processes. Acetyl-CoA is generated from citrate produced in the tricarboxylic acid (TCA) cycle by the metabolic enzyme ATP-citrate lyase [136]. However, the reuse of acetyl moieties has been proposed as an alternative mechanism to generate acetyl-groups [126]. Different acetyl transferases have been reported to transfer acetyl-groups to histones, but just one is active in human cells: Tip60. Tip60-dependent H4-acetylation leads to recruitment of MRN complex, BRCA1, Rad51 and 53BP1 [12]. The respective histone deacetylation is mediated by HDACs and depends on NAD+ as an important metabolic co-factor for these enzymes [137].

NAD+ is also an important cofactor for the activity of sirtuins (SIRTs) and of poly-(ADP-ribose) polymerases (PARPs). NAD+ can be produced either in the de novo pathway or in the salvage pathway: in the salvage pathway, nicotinamide phosphoribosyltransferase (Nampt) and nicotinamide mononucleotide adenylyltransferase (Nmnat) generate NAD+ from nicotinamide (NAM) and ATP, producing PRPP as a byproduct. In contrast, the de novo pathway uses tryptophan to generate nicotinic acid mononucleotide (NAMN) in seven steps. Nmnat then converts NAMN to nicotinic acid adenine dinucleotide (NAAD), while NAD synthase (NADS) converts NAAD to NAD [138]. SIRTs use NAD+ as a co-factor to catalyze deacetylation of lysine residues whereas PARPs exert important functions in the regulation of DNA repair, replication, transcription and also chromatin packaging, as reviewed in detail elsewhere [139,140].

Another factor influencing the DDR and DNA is the methylation of DNA or histones. Methionine adenosyltransferase (MAT) produces S-adenosylmethionine (SAM) in an enzymatic reaction utilizing methionine and ATP [141,142]. Specific methyl-transferases then use SAM as a co-substrate to place methyl-groups either on histone proteins or on the DNA [143,144]. Herein, histone methyltransferases (HMTs) transfer methyl-groups to histones, whereas DNA methyltransferases (DNMTs) transfer methyl-groups to DNA, respectively [141,142,145]. As an example for the regulatory action of histone methylation in the DDR, histone H3 lysin 9 trimethylation (H3K9me3) promotes ATM-dependent recruitment of Tip60 [146] and of 53BP1 [12,147], thereby supporting HRR.

Taken together, posttranslational modifications of DDR proteins, DNA and chromatin are essential to mount an effective DDR. Therefore, the generation of limiting substrates (acetyl-CoA, SAM, NAD+ and phosphate groups in the form of ATP) must be ensured in the repairing cell by the activity of basal metabolic pathways to fuel DSB repair [20], such as glycolysis, TCA cycle, electron transport chain, folate pathway and lipid metabolism (for an overview see Table 1).

## 4. Role of Akt in the Generation of Basic Metabolites with Importance to DDR

Akt is involved in the regulation of essential metabolic pathways that provide ATP, essential metabolites or both. We, thus, propose the regulation of metabolic pathways as a new facet of the Akt-signaling with potential relevance to DNA repair and therapy resistance on the molecular level. In the following paragraphs, we will highlight the multifaceted role of Akt in regulating metabolic pathways that are required to provide essential metabolites and energy for successful DDR and efficient DSB repair.

### 4.1. Role of Akt in Regulating the Antioxidant Defense

**Figure 4:**
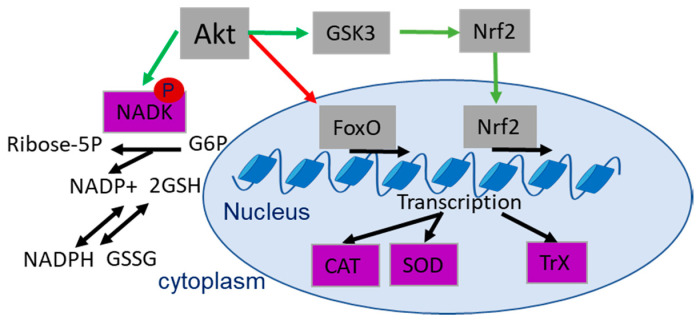
Schematic representation of antioxidant defense and Akt-dependent regulation of antioxidant enzymes (violet) related to radioresistance. Green arrows indicate Akt-dependent activation, red arrows show Akt dependent inhibition and black arrows show metabolic/transcriptional processes. Abbreviations: catalase (CAT), Forkhead-Box-Protein O3 (FoxO), glucose-6-phosphate (G6P), reduced glutathione (GSH), glycogen synthase kinase 3 (GSK3), Nicotinamide adenine dinucleotide (NADH), NAD kinase (NADK), nuclear factor erythroid 2-related factor 2 (Nrf2), superoxide dismutase (SOD), thioredoxin (TrX).

Various reports indicate a role of Akt in the regulation of the cellular antioxidant capacity and therapy outcome [148]: cells with active Akt tend to dispose of a high antioxidant capacity [110,149,150]. The involved processes include Nrf2-dependent induction of the thioredoxin (Trx) and glutathione-glutaredoxin (GrX) systems to enhance the production of NADPH [151] and GSH, respectively [108,148]. Furthermore, Akt directly phosphorylates its target protein NAD kinase (NADK) on Ser44, Ser46 and Ser48 [151]. Activating phosphorylation increases the NADK-dependent production of NADP+, and this is a prerequisite for GSH-dependent production of NADPH [151,152] (Figure 4). On the other hand, Akt-dependent phosphorylation of FoxO transcription factors reduces FoxO-dependent transcription of the antioxidant enzymes catalase (CAT) and mitochondrial superoxide dismutase (SOD2), thereby balancing antioxidant defense [18,110,153,154,155] (Figure 4).

### 4.2. Role of Akt in the Regulation of Glycolysis and Glutaminolysis

**Figure 5:**
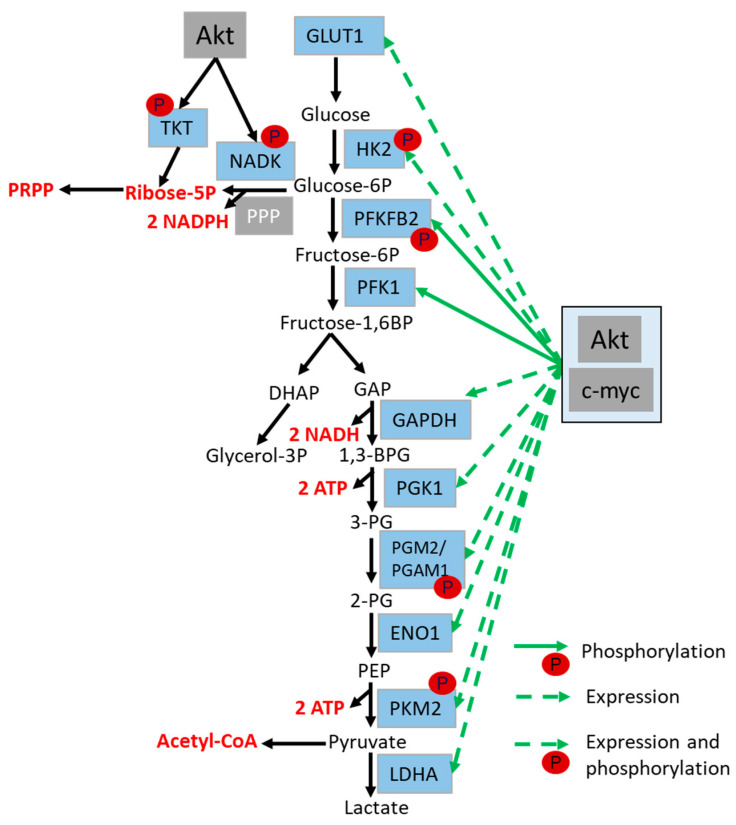
Schematic representation of major steps of glycolysis and direct and indirect regulation of glycolytic enzymes (in light blue) via Akt. Direct regulation of glycolysis occurs via Akt-mediated phosphorylation of glycolytic enzymes, whereas indirect regulation takes place via Akt-mediated c-myc activation. Akt and c-myc mediated activation are indicated with green arrows, the metabolic pathway is indicated with black arrows and the generated metabolites are marked in red. Abbreviations: 1,3-bisphosphogylcerate (1,3-BPG), Adenosine triphosphate (ATP), dihydroxyacetone phosphate (DHAP), enolase (ENO1), Glycerine aldehyde phosphate (GAP), Glyceraldehyde 3-phosphate dehydrogenase (GAPDH), Glucose transporter (GLUT), Hexokinase 2 (HK2), lactate dehydrogenase (LDHA), Nicotinamide adenine dinucleotide (NADH), NAD kinase (NADK), Nicotinamide adenine dinucleotide phosphate (NADPH), Phosphoenolpyruvic acid (PEP), pentose phosphate pathway (PPP), Phospho-fructo-kinase (PFK1), phosphoglycerate kinase 1 (PGK1), phosphoglycerate mutase 1 (PGAM1), transketolase (TKT).

Glucose, as the key molecule for the generation of energy and macromolecules, has to be imported into the cell (cytosol) by glucose transporters, or be produced from glycogen via glycogenolysis, respectively [156,157]. The first important step of glycolysis is the phosphorylation of glucose by hexokinase 2 (HK2) and the accompanied generation of glucose-6-phosphate (G6P). During glycolysis, G6P is further processed to pyruvate, thereby also leading to the production of NADH. Simultaneously, by shifting into the pentose phosphate pathway (PPP), G6P is used for the generation of NADPH and ribose-5-phosphate, and can thereby also feed nucleotide synthesis (Figure 5) [158,159,160].

It is widely accepted that Akt increases the expression of glucose transporter GLUT1 and GLUT4 via HIF1 and c-myc, to support glucose uptake in order to fuel glycolysis with relevance to radioresistance [161]. In addition, Akt stimulates cytosolic ATP production by regulating the expression and activity of different enzymes involved in glycolysis [48] (Figure 5).

For example, hexokinase 2 (HK2) is an important Akt target protein. Akt-mediated HK-2 phosphorylation increases the production of G6P and enhances glycolytic flux [162] through multiple mechanisms: (i) phosphorylation by Akt promotes HK2 binding to the mitochondria outer membrane, thereby enhancing the direct availability of ATP for the phosphorylation of glucose [162,163,164]; (ii) akt-dependent phosphorylation also promotes nuclear translocation of HK2 to repress the expression of genes of the carbohydrate metabolism [160,165,166]; (iii) finally, Akt-dependent activation of HIF1 promotes an increase in HK2 expression [163].

Akt also regulates glucose metabolism at the level of PFKFB2; PFKFB2-phosphorylation by Akt (at Ser483) promotes the production of fructose-2,6-bisphosphate, and thereby leads to the activation of phosphofructokinase (PFK1), which in turn promotes the generation of fructose-1,6-bisphosphate from fructose-6-phosphate (Figure 5) [167].

The phosphoglycerate mutase 1 (PGAM1) is another downstream target of Akt with important metabolic functions; however, PGAM1 not only regulates glycolysis (Figure 5), but also PPP and serine biosynthesis, and is associated with proliferation, metastasis and survival of cancer cells [168]. Of note, PGAM1 impacts dNTP pools by increasing the levels of ribose-5-phosphate and supports DSB end resection by stabilizing CTBP-interacting protein (CtIP) [169]. Thus, while PGAM1 activation improves HRR, PGAM1 inhibition sensitizes cancer cells to PARP inhibition by promoting a so-called “HR-ness” phenotype [169].

Finally, recent studies point to an Akt-dependent regulation of PKM2; PKM2 regulates the last step of glycolysis, the reaction of phosphoenolpyruvic acid (PEP) to ATP and pyruvate, which is then used for acetyl-CoA production or to fuel the TCA cycle in mitochondria (Figure 5) [170,171].

Another metabolite involved in the regulation of many of the mentioned metabolic processes is the amino acid glutamine. Glutamine is converted to glutamate and further to α-ketoglutarate, which can fuel the TCA cycle in the mitochondria in a process called glutaminolysis [172,173]. Furthermore, glutamine regulates mTORC1, autophagy and is involved in antioxidant production, nucleotide synthesis and fatty acid metabolism (reviewed in detail in [173]).

Akt regulates the import of glutamine into the cell through c-myc-mediated transcription and translation of the amino acid transporter type 2 (ASCT2/SLC1A5) [174,175]. The resulting increased import of glutamine supports increased TCA cycle activity, glucose metabolism, antioxidant defense, nucleotide synthesis and lipid synthesis and was at least in part mediated by Akt-dependent activation of mTORC1 [80,173]. Moreover, Akt might also contribute to the observed increase in c-myc activity and expression of the glutamine metabolizing enzyme glutaminase (GLS) [176]. In fact, c-myc is known to regulate the expression of different components of glucose and glutamine metabolism, e.g., HK2, PFK, alpha-enolase (ENO1), glyceraldehyde 3-phosphate dehydrogenase (GAPDH), phosphoglycerate kinase 1 (PGK1), lactate dehydrogenase (LDHA), PDK1, Glut1 and, as already mentioned, of GLS and ASCT2 [177].

Vice versa, a glycolytic enzyme, the alpha-enolase (ENO1), was found to regulate the PI3K/Akt pathway and thereby enhance proliferation and migration beyond its function in regulating glycolysis [178,179]. Another Akt-regulating metabolic enzyme is the cytosolic flavoprotein, NAD(P)H Quinone Dehydrogenase 1 (Nqo1) [180]. Nqo1 inhibits the Akt-antagonists PP2A and PTEN activity, which, in turn, could indirectly promote enhanced Akt activity [180]. Interestingly, in cells without Nqo1, Akt’s activity was inhibited and the expression of glycolysis and glutaminolysis genes was suppressed [180]. Of note, Nqo1 needs NADPH for the reduction of quinones, which are involved in the detoxification of radicals, suggesting an additional role in regulating the cellular antioxidant defense [180].

### 4.3. Role of Akt in the Regulation of the Mitochondrial Function

**Figure 6:**
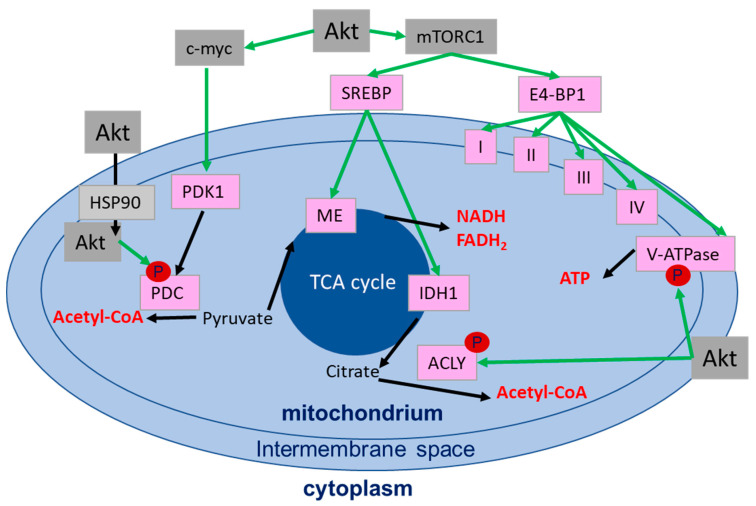
Schematic representation of the direct and indirect regulation of mitochondrial enzymes (in pink) by Akt. Akt-regulated activation is indicated with green arrows, metabolic processes are indicated by black arrows and the generated metabolites are shown in red. Abbreviations: ATP-citrate lyase (ACLY), heat shock protein 90 (HSP90), isocitrate dehydrogenase 1 (IDH1), malic enzyme (ME), mechanistic target of rapamycin/mammalian target of rapamycin (mTORC1), pyruvate dehydrogenase complex (PDC), pyruvate dehydrogenase kinase 1 (PDK1), sterol regulatory element binding proteins (SREBP).

Pyruvate generated as a result of glycolysis is converted by pyruvate dehydrogenase (PDH) to acetyl-CoA and glutamate (generated from glutamine) to fuel the TCA cycle in the mitochondria [181]. The complex reactions in the TCA cycle generate various metabolites involved in energy homeostasis, redox balance and macromolecule synthesis. These reactions are mediated among others by mitochondrial isocitrate dehydrogenase (IDH2), succinate dehydrogenase (SDH) and fumarate hydratase (FH) [181]. The generated reductive equivalents NADPH, NADH and FADH_2_, can be recycled/oxidized at the mitochondrial membrane by different respiratory complexes (complex I–V). These oxidation/reduction reactions generate an electron flux through the respiratory complexes towards molecular oxygen (ETC-chain) and a proton flux through the inner mitochondrial membrane. The proton gradient is then used by the ATP synthase (F0F1-ATPase) for generating ATP, a process called oxidative phosphorylation (Figure 6) [159,182].

**Figure 7:**
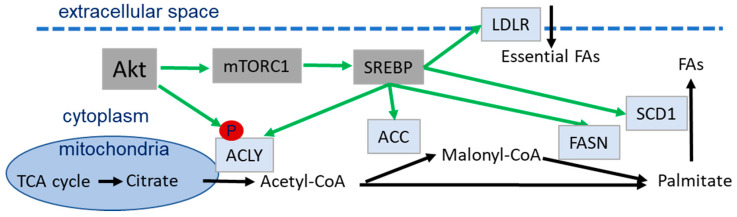
Schematic representation of fatty acid synthesis and Akt-mediated regulation of lipid metabolism via phosphorylation of its targets and SREBP-dependent expression of various proteins (in light blue). Green arrows indicate Akt-dependent activation, black arrows show the metabolic pathway. Abbreviations: acetyl-CoA carboxylase (ACC), ATP-citrate lyase (ACLY), fatty acid (FA), fatty acid synthase (FASN), low density lipoprotein receptor (LDLR) mechanistic target of rapamycin/mammalian target of rapamycin (mTORC1), stearoyl-coenzyme A desaturase 1 (SCD1), Sterol regulatory element binding proteins (SREBP).

Akt exerts multiple functions in the regulation of mitochondrial function and ATP production, though the molecular mechanisms require further definition (Figure 6): (i) it has been revealed that Akt translocates to and accumulates at the surface of mitochondria with support of the heat shock protein-90 (HSP90) [183]. PDK1 and mTORC2, two major activators of Akt, also translocate to the mitochondria, suggesting phosphorylation and activation of Akt at the mitochondria [184]. (ii) Improved ATP production as a result of active Akt had been linked to mTORC1- and 4E-BP1-dependent regulation of the respiratory complexes I, III and IV [185]. (iii) Further reports revealed an AMPK and Akt-dependent positive regulation of V-ATPase assembly [186] or an Akt-dependent phosphorylation of ATP-synthase inside the mitochondria [187], thereby enhancing ATP production. (iv) Other groups observed a role of Akt in phosphorylation of the PDH catalytic subunit PDH-E1α with impact on oxidative metabolism and acetyl-CoA production from pyruvate [188], or an interaction of mitochondrial Akt with PDH subunit PDHX in the pyruvate dehydrogenase complex (PDC), thereby regulating PDC activity [189]. In turn, PDC was observed to translocate to the nucleus for the de novo synthesis of acetyl-CoA from pyruvate, highlighting an alternative important route for the provision of acetyl-CoA for histone acetylation [190]. (v) Finally, Akt was shown to regulate the SREBP-mediated transcription of the malic enzyme (ME)1, ME2 and of all three IDH isoforms (1,2 and 3), thus influencing the TCA cycle activity and connecting mitochondrial metabolism with lipid metabolism (Figure 6 and Figure 7) [191,192].

Taken together, Akt’s role in regulating mitochondrial processes is complex und still not completely understood; however, the findings listed above document that besides the regulation of apoptosis though the mitochondrial pathway, Akt regulates mitochondrial metabolic functions to support survival and increase ATP production [193,194]. It is tempting to speculate that these processes might be particularly relevant for DSB repair in cancer cells with aberrant activation of Akt.

### 4.4. Role of Akt in Lipid Metabolism

Fatty acids are degraded via FAO in order to support energy generation in form of NADH, FADH_2_ (utilized in the electron transport chain) and acetyl-CoA (used in the TCA cycle) to produce ATP and NADPH (reviewed in [113]).

Akt’s role in regulating lipid metabolism is mostly exerted through mTORC1-dependent activation and inhibition of GSK3-dependent degradation of SREBP downstream of active Akt [48,80]. SREBPs are the key players in regulating fatty acid synthesis, both directly, by the expression of the required enzymes, and indirectly, by regulating regeneration of NADPH, which is an essential cofactor for fatty acid synthesis [191]. On the other hand, increasing levels of malonyl-CoA activate carnitine palmitoyltransferases (CPTs), which initiates FAO in the mitochondria (Figure 7) [191].

Akt-dependent activation of SREBP leads to the transcription of proteins involved in fatty acid synthesis, e.g., ATP citrate lyase (ACLY), acetyl-CoA carboxylase (ACC), fatty acid synthase (FASN), stearoyl-coenzyme A desaturase 1 (SCD1) and low density lipoprotein receptor (LDLR); moreover, it leads to the generation of NADPH, an essential co-factor in fatty acid production, and pathways required to convert acetate and glutamine into acetyl-CoA (Figure 7) [191,192,195,196].

In the cytosol, ACLY converts citrate to oxaloacetate (used in the TCA cycle) and acetyl-CoA, which can be used for the lipid synthesis by further conversion to malonyl-CoA by ACCs (Figure 7) [191,197]. On the one hand, malonyl-CoA and acetyl-CoA can be used to generate palmitate to provide the basic product for the production of fatty acids in a process regulated by fatty acid synthetase (FASN) (Figure 7) [191].

Furthermore, Akt can also regulate transcription of lipid metabolic proteins by FoxO and fat mass and obesity-associated protein (FTO) [198,199]. Similarly, the Akt-mTORC1 axis impacts lipogenesis by 40S ribosomal protein S6 (RPS6)-dependent posttranscriptional regulation of FASN ubiquitination, at least in HCC cell lines [200].

Generation of citrate during mitochondrial respiration as well as of glycerol-3P from glycolysis are essential substrates for lipid synthesis, underlining the complex interplay of different metabolic pathways involved in lipid metabolism and the key and multifaceted roles of Akt in balancing these processes from different angles [113,201].

Of note, fatty acids can be used to generate substrates with importance to the DDR, such as NADH, FADH_2_ and acetyl-CoA [191]. Moreover, Akt was found to phosphorylate the acetyl-CoA-generating enzyme ACLY, leading to its stabilization and increased activity in generating acetyl-CoA for lipid synthesis and histone modifications, respectively [197,202]. Furthermore, an indirect involvement of Akt in the regulation of acetyl-CoA carboxylase-alpha (ACCα) expression and phosphorylation by influencing ERK1/2 and ATM/AMPK has been described; these observations create an additional connection between cell metabolism and DNA repair [203].

### 4.5. Role of Akt in the Regulation of Nucleotide Synthesis

As described above (Section 3.2), the provision of purines and pyrimidines is required to fuel DNA synthesis during replication and DNA repair, respectively [20,116,118]. Importantly, the PI3K-Akt-mTORC1 pathway regulates various metabolic processes that are involved in generation of essential building blocks and co-substrates required for nucleotide generation, such as phospho-ribose pyrophosphate (PRPP), ATP and glutamine (Figure 3) [118]: (i) as outlined above (Section 4.2) Akt supports glycolytic activity at multiple levels, and thereby provides not only important co-factors for nucleotide synthesis, namely ATP, NADH and NADPH [161,162,163], but also glucose-6-phosphate (G6P) [116]. G6P is utilized in the PPP to generate ribose-5-phosphate, and thereby fuels the production of PRPP, an essential building block for de novo nucleotide synthesis or salvage [116,118,161,163]. Of note, Akt-dependent downregulation of AMPK also enhances the phosphorylation and activation of ribose-phosphate pyrophosphokinase 1/2 (PRPS1/2), and thus promotes increased conversion of ribose-5-P to PRPP to fuel nucleotide provision [118,204]. (ii) Akt promotes purine and pyrimidine synthesis, also through activating phosphorylation of its target protein transketolase (TKT) at Thr382: active TKT triggers the generation of ribose-5-P from fructose-5-P, and thus also supports PRPP generation [205]. (iii) Akt phosphorylates NADK, and this supports increased NADPH and tetrahydrofolate (THF) production [118,151]. (iv) Active AKT promotes mTORC1-dependent activation of S6K1 and the phosphorylation of CAD on S1859, thereby regulating the first step of pyrimidine synthesis and regulation of the folate pathway (for a review about mTOR signaling, see [80]) [118,206,207]. (v) Akt indirectly regulates the level of proteins involved in nucleotide synthesis, serine, glycine, folate-mediated one-carbon synthesis and glucose metabolism via c-myc, Nrf2 and ATF4 to fuel nucleotide synthesis at the level of metabolic enzymes and substrates [118,204,206,208].

### 4.6. Role of Akt in the Regulation of (Histone) Acetylation, Methylation and Ubiquitination

Akt regulates various metabolic processes that are involved in the production of metabolic co-factors with potential impact on DDR and DSB repair, e.g., acetyl-CoA and SAM, respectively. (i) As described in Section 3.3, acetyl-CoA is generated from citrate or pyruvate in the TCA cycle [209] and is used for the acetylation of proteins and chromatin during the DDR [202,210,211]. In this context, Akt is involved in the regulation of citrate and pyruvate production by glycolysis and mitochondrial metabolism, and in the generation of acetyl-CoA from citrate via ACLY or from pyruvate via PDC [48,190]. This Akt-dependent provision of acetyl-CoA can support acetylation processes including histone acetylation and lead to improved HRR [202]. (ii) Akt-dependent phosphorylation of GSK3β impacts phosphorylation of histone-acetylase Tip60, which is essential for its acetylase function [211]. (iii) The interaction between specific histone deacetylases, e.g., SIRT1 and HDAC1/3/6, and Akt allows an interactive and fast regulation of histone acetylation signals through the regulation of Akt’s activation state [212,213,214].

Beyond that, SAM functions as a methyl donor for histone and DNA methylation with relevance to the DDR and DSB repair: for example, methylation of H3K9 (H3K9me3) supports DSB repair [142]. In this context, the c-myc target protein methionine adenosyltransferase (MAT) catalyzes the generation of SAM from methionine and ATP [141,145]. Additionally, c-myc also regulates the transcription of the amino acid transporter ASCT2 to support the uptake of methionine into the cell [174,215]. Several reports point to a role of Akt in the regulation of histone methylation as follows: Akt-dependent phosphorylation led to the stabilization of the histone methyltransferase WHSC1 and resulted in enhanced prostate cancer metastasis downstream of mTORC2 [216]. In contrast, Akt-dependent phosphorylation of the histone demethylase KDM5A resulted in translocation of KDM5A from the nucleus to the cytoplasm, maintenance of enhanced histone H3K4 trimethylation, cell cycle progression, mitosis and DNA replication [217].

As stated above, the ubiquitination of proteins is also important for the recruitment of proteins involved in DSB repair and for ensuring repair dynamics, e.g., by mediating extraction of proteins from DSB repair complexes [127,218]. In this context, Akt has been reported to act as regulator of two E3 ubiquitin ligases, namely ring-finger protein 126 (RNF126) and breast cancer associated gene 2 (BCA2) [219]. RNF126 is involved in NHEJ repair by regulating Ku70/80, RNF168 and RNF8, whereas BCA2 regulates HRR through Rad51 and γH2AX [219]. Furthermore, the ubiquitin conjugating enzyme UBE2S is a direct target of Akt with potential relevance to improved DNA DSB repair [87]. Interestingly, Akt was shown to phosphorylate USP14, enhancing its deubiquitinating activity on K48 and K63 ubiquitin linkages, thereby promoting tumor development and cisplatin resistance as well as DSB repair dynamics over regulation of RNF168 mediated ubiquitination [220,221].

**Figure 8:**
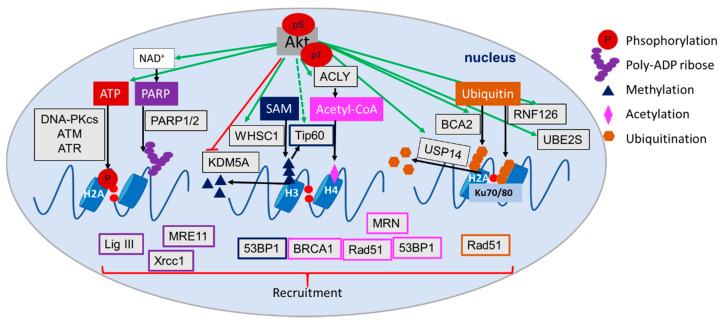
Schematic representation of important metabolites generated in glycolysis, pentose phosphate pathway (PPP), mitochondrial metabolism, lipid metabolism, SAM and NAD+ production with examples of Akt-dependent direct and indirect regulation of posttranslational modifications in the DNA damage response (DDR) and double strand break (DSB) repair. Metabolites indicated in different colors: ATP and phosphorylation in red, PARP and PARylation in dark violet, SAM and methylation in dark blue, acetyl-CoA and acetylation in light violet and ubiquitin and ubiquitination in orange; the downstream recruited proteins are marked with the same colors. Akt-regulated proteins are marked in light grey, green arrows indicate Akt-dependent activation, red arrows show Akt-dependent inhibition and black arrows indicate metabolic processes. Abbreviations: Tumor suppressor p53-binding protein 1 (53BP1), ATP citrate lyase (ACLY), ataxia telangiectasia mutated (ATM), ataxia telangiectasia and Rad3 related (ATR), breast cancer associated gene 2 (BCA2), Breast cancer type 1 susceptibility protein (BRCA1), DNA-dependent protein kinase, catalytic subunit (DNA-PKcs), Mre11, Rad50 and Nbs1 complex (MRN), Nicotinamide adenine dinucleotide (NAD+), poly-(ADP-ribose) polymerase (PARP), ring-finger protein 126 (RNF126), S-adenosylmethionine (SAM), Ubiquitin conjugating enzyme E2 S (UBE2S), X-ray repair cross-complementing protein 1 (Xrcc1).

Overall, these observations support the important and multifaceted roles of Akt in regulating the posttranslational modifications of DDR and DNA repair proteins and thereby of the dynamics of DNA repair processes. Herein, Akt directly and indirectly regulates the activity of metabolic enzymes, the production of metabolites as well as the expression, localization, stability or activity of epigenetic enzymes with relevance to the DDR and DSB repair (Figure 8).

## 5. Therapeutic Implications

The PI3K/Akt pathway is a key regulator of various signaling pathways. In the above sections, we highlighted the multifaceted role of active Akt in orchestrating cell survival, cell metabolism and DDR, including DSB repair as well as the documented and some assumed consequences for the cellular response to radiotherapy. The high frequency of an aberrant activation of the PI3K/Akt pathway in cancer cells and the observed consequences for cancer cell radiation resistance make Akt, its upstream regulators and specific downstream effectors interesting therapeutic targets for improving the therapy outcome. However, the role of Akt in inhibition and modulation of the response to genotoxic therapies/radiotherapy by regulating the respective metabolic pathways remains to be explored. Table 2 summarizes target proteins of Akt with potential relevance as therapeutic targets ordered by their activity in the particular pathway.

Various inhibitors of Akt-regulated enzymes functioning in the glycolysis pathway are already available. In fact, inhibitors of GLUTs, HK2, PGAM1, PKM2, LDHA and PDK have been tested or are tested in preclinical investigations (as reviewed in [222]). As an example, the upregulated expression of the glycolytic enzyme PGAM1 in many cancers and its impact on cancer prognosis make this enzyme an interesting therapeutic target [223,224]. Intriguingly, beyond its function in glycolysis, PGAM1 also impacts HRR by regulating the dNTP pool [169]; thus, a combination therapy targeting both PGAM1 and PARP has been proposed. Beyond that, inhibition of the Akt-regulated PDK-PDH axis by using PDK-inhibitors such as dichloroacetate (DCA) induces cell death in cancer cells and exerts promising antineoplastic actions in laboratory models in different cancers [225,226]. Another emerging therapeutic target is PKM2, which is upregulated in erlotinib resistant tumor cells as a consequence of aberrant Akt activation [171].

As outlined above, Akt is also involved in the regulation of mitochondrial function. Of note, drugs targeting the mitochondria and the mitochondrial oxidative phosphorylation emerge as promising targets in cancer therapy [227,228,229]. Among those, especially metformin, rotenone, malonate, atovaquone, hydrocortisone and oligomycin are promising examples (for a more detailed discussion see [227,228]). However, the most interesting target in the TCA cycle is IDH1/2: IDH1/2 is frequently mutated in aggressive cancers and contributes to various aspects of malignant growth, including also epigenetic regulation of DSB repair [22,230]. Drugs targeting both IDH isoforms have already been approved for cancer treatment [231,232]. It is tempting to speculate, that Akt’s role in regulating these metabolic processes might be particularly promising in tumors with aberrant Akt activation.

Akt also regulates fatty acid synthesis and thereby lipid availability. Lipids are essential molecules for cancer progression, promoting the growth and survival of cancer cells [233]. Therefore, the opportunities for targeting lipid metabolism in cancer therapy are under current investigation. In fact, SREBP itself and all expressed proteins in lipid metabolism (ACCs, ACLY, FASN, LDLR, etc.) constitute potential cancer targets, and the respective inhibitors are already tested in preclinical and phase 1 trials [196,233]. Of note, Akt regulates the transcription factor SREBP that controls the expression of nearly all enzymes involved in lipid metabolism [191,195]. Furthermore, the Akt target protein ACLY links glycolysis and lipid synthesis by providing acetyl-CoA, the starting molecule for FAO. However, ACLY is also involved in histone acetylation and DSB repair, making this key metabolic enzyme of cancer signaling an attractive therapeutic target in combination with radiotherapy [202]. Already in the past, many inhibitors targeting ACLY have been investigated in vitro and in vivo for different cancer types, including hydroxycitrate, radicicol, deoxychlorate, vanadate and others (for a review about the mechanistical action, see [234,235]). However, it remains to be explored how pharmacologic inhibition of aberrant activation of Akt impacts DSB repair and radiation sensitivity through modulating ACLY’s nuclear function.

As discussed in this review, the antioxidant system is frequently involved in therapy resistance [236]. In fact, drugs targeting the thioredoxin and glutathione system (Trx, TrxR and GSH) have been identified as promising targets for cancer therapy, especially in combination with radiotherapy [24,112,236,237]. The same holds true for the antioxidant enzyme catalase [153]. Again, the impact of therapeutic inhibition of Akt on the antioxidant capacity of cancer cells and the resulting effect on radiation resistance needs to be further explored.

Akt also regulates NADPH and NADH producing processes. The production and regeneration of both metabolites are potential targets for cancer therapy: (i) NADPH is involved in antioxidant defense; (ii) NADH and NADPH are needed for OxPhos, the production of PARP and as cofactors for several metabolic enzymes [238]. Current investigations explore the use inhibitors targeting essential enzymes for NAD and NADP generation in cancer therapy, such as nicotinamide phosphoribosyltransferase (Nampt) and NADK [238,239]. However, it remains to be explored if inhibition of Akt will impact radiosensitivity through modulating levels of NADPH, NADH, or both.

Further studies investigate if inhibition of enzymes catalyzing posttranslational modifications of DNA and histones that are considered to be essential for DNA DSB repair, interfere with the DNA repair and improve eradication of cancer cells [218,240,241,242,243,244,245,246,247]. As the most prominent example, PARP inhibitors are already tested in clinical studies as mono- or combination therapy [240,241]. Further potential cancer targets involved in posttranslational regulation of the DDR include histone methyltransferases, lysine specific demethylases, HATs and HDACs [243,244,245,246,247], as well as ubiquitination and deubiquitylation enzymes and ubiquitin-specific proteases such as the Akt phosphorylation target USP14 [218,220,248,249,250]. Interestingly, deregulation of the PI3K-Akt pathway is frequently observed in tumors developing resistance to treatment with PARP inhibitors [242]. This observation suggests a potential contribution of Akt to acquired resistance to PARP-inhibition, potentially also involving mitochondrial protection against oxidative stress [242].

The relevance of drugs targeting protein and chromatin modifications is already well established (and discussed in [246,247]). However, the role of Akt in regulating those proteins influencing ubiquitination (via UBE2S [87,251]), acetylation (via ACLY [202]) or methylation processes (via SAM [174,215]) has not yet been considered. We, thus, propose to specifically investigate the role of these proteins in tumors with Akt-mediated resistance.

**Table 2:** Downstream targets of Akt as (potential) therapeutic targets.

Pathway		Targets	References
TCA & OxPhos	Respiratory chain	Complex I–V	[227,229]
		Cytochrome bc1 complex	[228]
		Cytochrome c oxidase	[228]
	TCA cycle	IDH	[231,232]
		Citrate	[252]
Glycolysis		GLUTs	[222]
		HKs	[222]
		PFK	[222]
		PGAM1	[222,224]
		PKM2	[222]
		LDHA	[222]
		MCTs	[222]
		PDK	[222,225,226]
		PDHA	[225,226]
Lipid metabolism		ACLY	[234,235]
		ACC	[191]
		FASN	[191]
		SCD1	[191]
		LDLR	[191]
Nucleotide pools		dNTP pool	[119,253]
Antioxidant system	Thioredoxin system	TrX	[236,237]
		TrxR	[236,237]
		ASK1	[237]
		ROS	[237]
		GSH	[237]
	NADPH production	NADK	[239]
		NAMPT	[238]
Epigentic modifications	Acetylation	HATs	[246]
		HDACs	[243,245,246]
	Methylation	Histone (lys) methyltransferases	[246,247]
		protein arginine methyltransferases	[246,247]
		histone lysine demethylases	[246,247]
		DNA methyltransferases	[246]
	Ubiquitination	E1 enzyme	[218,249,250]
		E2 enzyme	[218,249,250]
		E3 ligases	[218,249,250]
		Deubiquitinases (DUBs)	[218,220,248,249,250]
	Parylation	PARP inhibitors	[240,241]

## 6. Conclusions and Outlook

**Figure 9:**
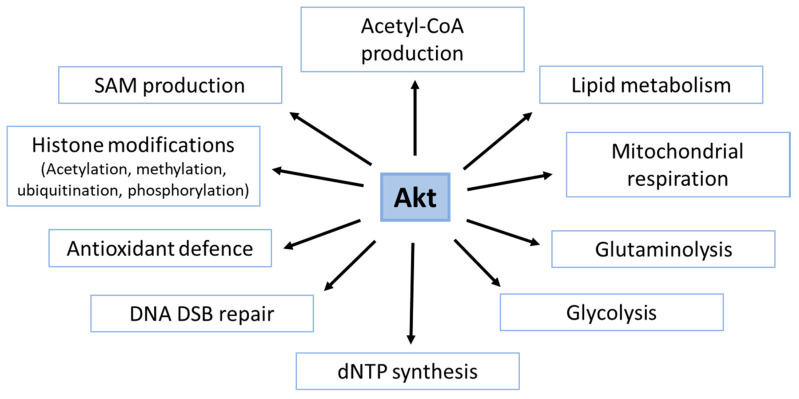
Schematic representation of Akt regulated pathways involved in DNA damage response including DSB repair.

In conclusion, signaling through the PI3K/Akt pathway, and particularly through Akt, plays complex and interconnected roles in the regulation of the DDR and DSB repair; it involves both the interaction of Akt with components of the DDR as well as the regulatory role of Akt in cell metabolism (Figure 9). It is assumed that Akt’s ability to balance signaling processes in the DDR with metabolic processes supports its role as a key regulator of cell survival, particularly under stress conditions, and thereby, also enhances cancer cell resistance to genotoxic therapies. In this context, Akt-dependent phosphorylation of effector proteins influences not only the activity of specific proteins in the DDR but also the activity of important metabolic regulators such as mTORC1, GSK3 and AMPK; beyond that, Akt also regulates transcription factors with an impact on cell metabolism and cell survival such as SREBP, myc, FoxO, HIF1 and Nrf2.

In the present review, we specifically highlight the modulation of cancer metabolism as an emerging novel facet of Akt-mediated therapy resistance; in fact, accumulating evidence indicates that Akt is either directly or indirectly involved in the regulation of major metabolic processes such as glycolysis, glutaminolysis, PPP, mitochondrial metabolism, lipid metabolism, nucleotide synthesis and antioxidant defense. These pathways provide metabolites and co-factors essential to the protection against radiation-induced cell damage and execution of DDR and DSB repair, respectively. In addition, Akt regulates proteins involved in DNA methylation as well as histone modifications (e.g., acetylation, ubiquitination, methylation) to sustain the DDR beyond regulating the processes producing the metabolites required for these modifications.

Importantly, Akt-dependent provision of essential metabolites and co-factors required for the detoxification of ROS and the execution of DDR and DSB, including epigenetic regulation of chromatin structure, creates potential vulnerabilities for improving the outcome of radiotherapy in cancers with aberrant Akt activity. In summary, the majority of the Akt-dependent metabolic and DNA repair processes mentioned in this review represent potential target structures for therapeutic intervention (Figure 9). The advantage of using either drugs inhibiting Akt directly or specific downstream effector proteins of Akt remains to be explored.

## 7. Methods

Studies in English were identified via searching electronic databases, e.g., Pubmed, Web of Science with the following keywords: Akt, Akt target, phosphorylation, PI3K, radiotherapy, ionizing radiation, therapy resistance, DNA damage response, DDR, DNA repair, DSB, cancer metabolism, metabolites, chromatin modifications, antioxidant defense. Studies and reviews that focused on ionizing radiation Akt and/or metabolism were included. Publications focusing on novel cancer therapies, such as hormonal therapy, were excluded. To be more reliable, conclusions from different publications were cross examined. Unpublished materials were not included in this review.

## Figures and Tables

**Table 1:** Generated metabolites in cellular pathways relevant to the DDR. Abbreviations: α-ketoglutarate (αKG) Adenosine triphosphate (ATP), Flavin adenine dinucleotide (FADH_2_), reduced glutathione (GSH), Nicotinamide adenine dinucleotide (NADH), Nicotinamide adenine dinucleotide phosphate (NADPH), Nucleotide triphosphate (NTP), phospho-ribose pyrophosphate (PRPP), S-adenosylmethionine (SAM), tricarboxylic acid cycle (TCA).

Antioxidant Defense	Nucleotide Synthesis	Glycolysis	Glutaminolysis	SAM Production	TCA & OxPhos	Lipid Metabolism
NADPH	PRPP	Pyruvate	αKG	Met	ATP	Acetyl-CoA
GSH	NADH	Lactate	ATP	SAM	NADH	Malonyl-CoA
	NADPH	NADH			FADH_2_	Palmitate
	Pyrimidine	ATP			Acetyl-CoA	Fatty acids
	Purine					
	dNTPs					

## Abbreviations

1,3-BPG	1,3-bisphosphogylcerate
53BP1	Tumor suppressor p53-binding protein 1
ACC	acetyl-CoA carboxylase
ACLY	ATP citrate lyase
AMP	adenosine monophosphate
AMPK	AMP-activated protein kinase
ASCT2	amino acid transporter type 2
ATM	ataxia telangiectasia mutated
ATP	Adenosine triphosphate
ATR	ataxia telangiectasia and Rad3 related
BCA2	breast cancer associated gene 2
BRCA1	Breast cancer type 1 susceptibility protein
BRCC	BRCA1/BRCA2-containing complex
CAD	carbamoyl-phosphate synthetase
CAT	Catalase
CPT	carnitine palmitoyltransferase
CtIP	CTBP-interacting protein
DDR	DNA damage response
DNA-PKcs	DNA-dependent protein kinase, catalytic subunit
DNMT	DNA methyltransferase
DSB	Double strand break
DUB	deubiquitinase
EMSY	BRCA2-interacting transcriptional repressor
ENO1	enolase
FADH_2_	Flavin adenine dinucleotide
FAO	Fatty acid oxidation
FASN	fatty acid synthase
DHAP	dihydroxyacetone phosphate
FH	fumarate hydratase
FoxO	Forkhead-Box-Protein O3
FTO	fat mass and obesity-associated protein
G6P	glucose-6-phosphate
GAP	Glycerine aldehyde phosphate
GAPDH	Glyceraldehyde 3-phosphate dehydrogenase
GLS	Glutaminase
GLUT	Glucose transporter
GMP	guanine monophosphate
GPCR	G-protein coupled receptors
GrX	glutathione-glutaredoxin/glutathione peroxidase
GSH	reduced glutathione
GSK3	glycogen synthase kinase 3
GTP	Guanine triphosphate
HAT	Histone acetyltransferase
HDAC	Histone deacetylase
HIF1	Hypoxia inducible factor 1
HK2	Hexokinase 2
HMT	Histone methyltransferase
HRR	homologous recombination repair
HSP90	heat shock protein-90
IDH	isocitrate dehydrogenase
INPP4B	Inositol polyphosphate 4-phosphatase type II
LDHA	lactate dehydrogenase
LDLR	low density lipoprotein receptor
MAT	methionine adenosyltransferase
MDM2	Mouse double minute 2 homolog
MERIT40	mediator of Rap80 interactions and targeting 40 kDa
MRN	Mre11, Rad50 and Nbs1 complex
MTHFD	formyltetrahydrofolate synthetase
MTHFD1/2	Methylenetetrahydrofolate Dehydrogenase
mTOR	mechanistic target of rapamycin/mammalian target of rapamycin
NAAD	nicotinic acid adenine dinucleotide
NAD+/H	Nicotinamide adenine dinucleotide
NADK	NAD kinase
NADPH	Nicotinamide adenine dinucleotide phosphate
NADS	NAD synthase
NAM	nicotinamide
NAMN	nicotinic acid mononucleotide
Nampt	nicotinamide phosphoribosyltransferase
NAMPT	Nicotinamide phosphoribosyltransferase
NHEJ	non-homologous end joining
Nmnat	nicotinamide mononucleotide adenylyltransferase
Nqo1	cytosolic flavoprotein, NAD(P)H Quinone Dehydrogenase 1
Nrf2	nuclear factor erythroid 2-related factor 2
NTP	Nucleotide triphosphate
OGDH	oxoglutarate dehydrogenase
OxPhos	oxidative phosphorylation
PARP	poly-(ADP-ribose) polymerase
PDC	pyruvate dehydrogenase complex
PDH	pyruvate dehydrogenase
PDK1	Pyruvate dehydrogenase kinase 1
PDK1	phosphoinositide-dependent kinase 1
PEP	Phosphoenolpyruvic acid
PFK	Phospho-fructo-kinase
PGAM1	phosphoglycerate mutase 1
PGK1	phosphoglycerate kinase 1
PH	pleckstrin homology
PHLPP1/2	PH domain and leucine rich repeat protein phosphatase 1
PI3K	Phosphatidyl-inositol-3-kinase
PKM2	Pyruvate kinase M2
PPP	pentose phosphate pathway
PRPP	phospho-ribose pyrophosphate
PRPS1/2	ribose-phosphate pyrophosphokinase 1/2
PTEN	phosphatase and tensin homologue
Rap80	receptor-associated protein 80
RNF126	Ring finger protein 126
ROS	reactive oxygen species
RTK	Receptor tyrosine kinase
S6K	S6 kinase
SAM	S-adenosylmethionine
SCD1	stearoyl-coenzyme A desaturase 1
SDH	succinate dehydrogenase
SIRT	Sirtuin
SOD	superoxide dismutase
SREBP	Sterol regulatory element binding proteins
TCA	tricarboxylic acid cycle
THF	tetrahydrofolate
TKT	transketolase
TNKS	tankerase
TrX	thioredoxin
TSC2	tuberous sclerosis complex 2
UBE2S	Ubiquitin conjugating enzyme E2 S
UMP	uridine monophosphate
XLF	XRCC4-like factor
XRCC1	X-ray repair cross-complementing protein 1

## References

[1] Mladenov E., Magin S., Soni A., Iliakis G. (2013). DNA Double-Strand Break Repair as Determinant of Cellular Radiosensitivity to Killing and Target in Radiation Therapy. Front. Oncol..

[2] Mehta A., Haber J.E. (2014). Sources of DNA double-strand breaks and models of recombinational DNA repair. Cold Spring Harb. Perspect. Biol..

[3] Mavragani I.V., Nikitaki Z., Kalospyros S.A., Georgakilas A.G. (2019). Ionizing radiation and complex DNA damage: From prediction to detection challenges and biological significance. Cancers.

[4] Lieber M.R. (2010). The mechanism of double-strand DNA break repair by the nonhomologous DNA end-joining pathway. Annu. Rev. Biochem..

[5] Sen T., Gay C.M., Byers L.A. (2018). Targeting DNA damage repair in small cell lung cancer and the biomarker landscape. Transl. Lung Cancer Res..

[6] Jackson S.P., Bartek J. (2009). The DNA-damage response in human biology and disease. Nature.

[7] Iliakis G., Murmann T., Soni A. (2015). Alternative end-joining repair pathways are the ultimate backup for abrogated classical non-homologous end-joining and homologous recombination repair: Implications for the formation of chromosome translocations. Mutat. Res. Genet. Toxicol. Environ. Mutagen..

[8] Mladenov E., Iliakis G. (2011). Induction and repair of DNA double strand breaks: The increasing spectrum of non-homologous end joining pathways. Mutat. Res. Fundam. Mol. Mech. Mutagen..

[9] Jachimowicz R.D., Goergens J., Reinhardt H.C. (2019). DNA double-strand break repair pathway choice—From basic biology to clinical exploitation. Cell Cycle.

[10] Lukas J., Lukas C., Bartek J. (2011). More than just a focus: The chromatin response to DNA damage and its role in genome integrity maintenance. Nat. Cell Biol..

[11] Soria G., Polo S.E., Almouzni G. (2012). Prime, Repair, Restore: The Active Role of Chromatin in the DNA Damage Response. Mol. Cell.

[12] Murr R., Loizou J.I., Yang Y.G., Cuenin C., Li H., Wang Z.Q., Herceg Z. (2006). Histone acetylation by Trrap-Tip60 modulates loading of repair proteins and repair of DNA double-strand breaks. Nat. Cell Biol..

[13] Xu Y., Ayrapetov M.K., Xu C., Gursoy-yuzugullu O., Hu Y., Price B.D. (2012). H2AZ controls DSB repair. Mol. Cell.

[14] Smeenk G., van Attikum H. (2013). The Chromatin Response to DNA Breaks: Leaving a Mark on Genome Integrity. Annu. Rev. Biochem..

[15] Price B.D., D’Andrea A.D. (2013). Chromatin remodeling at DNA double-strand breaks. Cell.

[16] Shia L., Oberdoerffer P. (2012). Chromatin dynamics in DNA double-strand break repair. Biochim. Biophys. Acta.

[17] Matschke V., Theiss C., Matschke J. (2019). Oxidative stress: The lowest common denominator of multiple diseases. Neural Regen. Res..

[18] Zhao Y., Hu X., Liu Y., Dong S., Wen Z., He W., Zhang S., Huang Q., Shi M. (2017). ROS signaling under metabolic stress: Cross-talk between AMPK and AKT pathway. Mol. Cancer.

[19] Efimova E.V., Takahashi S., Shamsi N.A., Wu D., Labay E., Ulanovskaya O.A., Weichselbaum R.R., Kozmin S.A., Kron S.J. (2015). Linking Cancer Metabolism to DNA Repair and Accelerated Senescence. Mol. Cancer Res..

[20] Turgeon M.O., Perry N.J.S., Poulogiannis G. (2018). DNA damage, repair, and cancer metabolism. Front. Oncol..

[21] van Vugt M.A.T.M. (2017). Shutting down the power supply for DNA repair in cancer cells. J. Cell Biol..

[22] Xiang K., Jendrossek V., Matschke J. (2020). Oncometabolites and the response to radiotherapy. Radiat. Oncol..

[23] Bennett C.B., Lewis A.L., Baldwin K.K., Resnick M.A. (1993). Lethality induced by a single site-specific double-strand break in a dispensable yeast plasmid. Proc. Natl. Acad. Sci. USA.

[24] Matschke J., Riffkin H., Klein D., Handrick R., Lüdemann L., Metzen E., Shlomi T., Stuschke M., Jendrossek V. (2016). Targeted Inhibition of Glutamine-Dependent Glutathione Metabolism Overcomes Death Resistance Induced by Chronic Cycling Hypoxia. Antioxid. Redox Signal..

[25] Jutten B., Rouschop K.M.A. (2014). EGFR signaling and autophagy dependence for growth, survival, and therapy resistance. Cell Cycle.

[26] Jiang H., Wang H., De Ridder M. (2018). Targeting antioxidant enzymes as a radiosensitizing strategy. Cancer Lett..

[27] Toulany M. (2019). Targeting DNA double-strand break repair pathways to improve radiotherapy response. Genes.

[28] Pilié P.G., Tang C., Mills G.B., Yap T.A. (2019). State-of-the-art strategies for targeting the DNA damage response in cancer. Nat. Rev. Clin. Oncol..

[29] Trenner A., Sartori A.A. (2019). Harnessing DNA Double-Strand Break Repair for Cancer Treatment. Front. Oncol..

[30] Biau J., Chautard E., Verrelle P., Dutreix M. (2019). Altering DNA repair to improve radiation therapy: Specific and multiple pathway targeting. Front. Oncol..

[31] Hanahan D., Weinberg R.A. (2011). Hallmarks of cancer: The next generation. Cell.

[32] Matschke J., Wiebeck E., Hurst S., Rudner J., Jendrossek V. (2016). Role of SGK1 for fatty acid uptake, cell survival and radioresistance of NCI-H460 lung cancer cells exposed to acute or chronic cycling severe hypoxia. Radiat. Oncol..

[33] Hlouschek J., Hansel C., Jendrossek V., Matschke J. (2018). The mitochondrial citrate carrier (SLC25A1) sustains redox homeostasis and mitochondrial metabolism supporting radioresistance of cancer cells with tolerance to cycling severe hypoxia. Front. Oncol..

[34] Kass E.M., Moynahan M.E., Jasin M. (2016). When Genome Maintenance Goes Badly Awry. Mol. Cell.

[35] Farmer H., McCabe H., Lord C.J., Tutt A.H.J., Johnson D.A., Richardson T.B., Santarosa M., Dillon K.J., Hickson I., Knights C. (2005). Targeting the DNA repair defect in BRCA mutant cells as a therapeutic strategy. Nature.

[36] Ashworth A., Lord C.J. (2018). Synthetic lethal therapies for cancer: what’s next after PARP inhibitors?. Nat. Rev. Clin. Oncol..

[37] McLornan D.P., List A., Mufti G.J. (2014). Applying synthetic lethality for the selective targeting of cancer. N. Engl. J. Med..

[38] Lord C.J., Ashworth A. (2017). PARP Inhibitors: The First Synthetic Lethal Targeted Therapy. Science.

[39] Stadler J., Richly H. (2017). Regulation of DNA repair mechanisms: How the chromatin environment regulates the DNA damage response. Int. J. Mol. Sci..

[40] Zernickel E., Sak A., Riaz A., Klein D., Groneberg M., Stuschke M. (2019). Targeting of BRM sensitizes BRG1-mutant lung cancer cell lines to radiotherapy. Mol. Cancer Ther..

[41] Riaz M.A., Sak A., Erol Y.B., Groneberg M., Thomale J., Stuschke M. (2019). Metformin enhances the radiosensitizing effect of cisplatin in non-small cell lung cancer cell lines with different cisplatin sensitivities. Sci. Rep..

[42] Bayo J., Tran T.A., Wang L., Peña-Llopis S., Das A.K., Martinez E.D. (2018). Jumonji Inhibitors Overcome Radioresistance in Cancer through Changes in H3K4 Methylation at Double-Strand Breaks. Cell Rep..

[43] Scanlon S.E., Glazer P.M. (2015). Multifaceted control of DNA repair pathways by the hypoxic tumor microenvironment. DNA Repair (Amst.).

[44] Kaplan A.R., Glazer P.M. (2020). Impact of hypoxia on DNA repair and genome integrity. Mutagenesis.

[45] Chan N., Koritzinsky M., Zhao H., Bindra R., Glazer P.M., Powell S., Belmaaza A., Wouters B., Bristow R.G. (2008). Chronic hypoxia decreases synthesis of homologous recombination proteins to offset chemoresistance and radioresistance. Cancer Res..

[46] Vivanco I., Sawyers C.L. (2002). The phosphatidylinositol 3-kinase-AKT pathway in humancancer. Nat. Rev. Cancer.

[47] Brognard J., Clark A.S., Ni Y., Dennis P.A. (2001). Akt/protein kinase B is constitutively active in non-small cell lung cancer cells and promotes cellular survival and resistance to chemotherapy and radiation. Cancer Res..

[48] Hoxhaj G., Manning B.D. (2019). The PI3K–AKT network at the interface of oncogenic signalling and cancer metabolism. Nat. Rev. Cancer.

[49] Fruman D.A., Rommel C. (2014). PI3K and cancer: Lessons, challenges and opportunities. Nat. Rev. Drug Discov..

[50] Manning B.D., Toker A. (2017). AKT/PKB Signaling: Navigating the Network. Cell.

[51] Szymonowicz K., Oeck S., Malewicz N., Jendrossek V. (2018). New Insights into Protein Kinase B/Akt Signaling: Role of Localized Akt Activation and Compartment-Specific Target Proteins for the Cellular Radiation Response. Cancers.

[52] Toulany M., Rodemann H.P. (2015). Phosphatidylinositol 3-kinase/Akt signaling as a key mediator of tumor cell responsiveness to radiation. Semin. Cancer Biol..

[53] Avan A., Narayan R., Giovannetti E., Peters G.J. (2016). Role of Akt signaling in resistance to DNA-targeted therapy. World J. Clin. Oncol..

[54] Turner K.M., Sun Y., Ji P., Granberg K.J., Bernard B., Hu L., Cogdell D.E., Zhou X., Yli-Harja O., Nykter M. (2015). Genomically amplified Akt3 activates DNA repair pathway and promotes glioma progression. Proc. Natl. Acad. Sci. USA.

[55] Gol T.M., Rodemann H.P., Dittmann K. (2019). Depletion of Akt1 and Akt2 impairs the repair of radiation-induced DNA double strand breaks via homologous recombination. Int. J. Mol. Sci..

[56] Mueck K., Rebholz S., Harati M.D., Rodemann H.P., Toulany M. (2017). Akt1 stimulates homologous recombination repair of DNA double-strand breaks in a Rad51-dependent manner. Int. J. Mol. Sci..

[57] Bozulic L., Surucu B., Hynx D., Hemmings B.A. (2008). PKBα/Akt1 Acts Downstream of DNA-PK in the DNA Double-Strand Break Response and Promotes Survival. Mol. Cell.

[58] Toulany M., Kehlbach R., Florczak U., Sak A., Wang S., Chen J., Lobrich M., Rodemann H.P. (2008). Targeting of AKT1 enhances radiation toxicity of human tumor cells by inhibiting DNA-PKcs-dependent DNA double-strand break repair. Mol. Cancer Ther..

[59] Vanhaesebroeck B., Guillermet-Guibert J., Graupera M., Bilanges B. (2010). The emerging mechanisms of isoform-specific PI3K signalling. Nat. Rev. Mol. Cell Biol..

[60] Kuo Y.C., Huang K.Y., Yang C.H., Yang Y.S., Lee W.Y., Chiang C.W. (2008). Regulation of phosphorylation of Thr-308 of Akt, cell proliferation, and survival by the B55α regulatory subunit targeting of the protein phosphatase 2A holoenzyme to Akt. J. Biol. Chem..

[61] Gao T., Furnari F., Newton A.C. (2005). PHLPP: A phosphatase that directly dephosphorylates Akt, promotes apoptosis, and suppresses tumor growth. Mol. Cell.

[62] Ferrari E., Bruhn C., Peretti M., Cassani C., Carotenuto W.V., Elgendy M., Shubassi G., Lucca C., Bermejo R., Varasi M. (2017). PP2A Controls Genome Integrity by Integrating Nutrient-Sensing and Metabolic Pathways with the DNA Damage Response. Mol. Cell.

[63] Chen C.Y., Chen J., He L., Stiles B.L. (2018). PTEN: Tumor suppressor and metabolic regulator. Front. Endocrinol. (Lausanne).

[64] Guertin D.A., Stevens D.M., Thoreen C.C., Burds A.A., Kalaany N.Y., Moffat J., Brown M., Fitzgerald K.J., Sabatini D.M. (2006). Ablation in Mice of the mTORC Components raptor, rictor, or mLST8 Reveals that mTORC2 Is Required for Signaling to Akt-FOXO and PKCα, but Not S6K1. Dev. Cell.

[65] Yang G., Murashige D.S., Humphrey S.J., James D.E. (2015). A Positive Feedback Loop between Akt and mTORC2 via SIN1 Phosphorylation. Cell Rep..

[66] Persad S., Attwell S., Gray V., Mawji N., Deng J.T., Leung D., Yan J., Sanghera J., Walsh M.P., Dedhar S. (2001). Regulation of protein kinase B/Akt-serine 473 phosphorylation by integrin-linked kinase: Critical roles for kinase activity and amino acids arginine 211 and serine 343. J. Biol. Chem..

[67] Oeck S., Al-Refae K., Riffkin H., Wiel G., Handrick R., Klein D., Iliakis G., Jendrossek V. (2017). Activating Akt1 mutations alter DNA double strand break repair and radiosensitivity. Sci. Rep..

[68] Guinea Viniegra J., Martínez N., Modirassari P., Hernández Losa J., Parada Cobo C., Sánchez-Arévalo Lobo V.J., Aceves Luquero C.I., Álvarez-Vallina L., Ramón Y., Cajal S. (2005). Full activation of PKB/Akt in response to insulin or ionizing radiation is mediated through ATM. J. Biol. Chem..

[69] Shiga S., Murata Y., Hashimoto T., Urushihara Y., Fujishima Y., Kudo K., Sonohara Y., Kurusu M., Takeda K., Jingu K. (2020). DNA-PKcs is activated under nutrient starvation and activates Akt, MST1, FoxO3a, and NDR1. Biochem. Biophys. Res. Commun..

[70] Khalil A., Morgan R.N., Adams B.R., Golding S.E., Dever S.M., Rosenberg E., Povirk L.F., Valerie K. (2011). ATM-dependent ERK signaling via AKT in response to DNA double-strand breaks. Cell Cycle.

[71] Maehama T., Dixon J.E. (1998). The Tumor Suppressor, PTEN/MMAC1, Dephosphorylates the Lipid Second Messenger, Phosphatidylinositol 3,4,5-Trisphosphate. J. Biol. Chem..

[72] Agoulnik I.U., Hodgson M.C., Bowden W.A., Ittmann M.M. (2011). INPP4B: The New Kid on the PI3K Block INPP4b structure ANd fuNctIoN. Oncotarget.

[73] Gewinner C., Wang Z.C., Richardson A., Teruya-Feldstein J., Etemadmoghadam D., Bowtell D., Barretina J., Lin W.M., Rameh L., Salmena L. (2009). Evidence that Inositol Polyphosphate 4-Phosphatase Type II Is a Tumor Suppressor that Inhibits PI3K Signaling. Cancer Cell.

[74] Datta S.R., Brunet A., Greenberg M.E. (1999). Cellular survival: A play in three akts. Genes Dev..

[75] Maurer U., Charvet C., Wagman A.S., Dejardin E., Green D.R. (2006). Glycogen synthase kinase-3 regulates mitochondrial outer membrane permeabilization and apoptosis by destabilization of MCL-1. Mol. Cell.

[76] Sears R., Nuckolls F., Haura E., Taya Y., Tamai K., Nevins J.R. (2000). Multiple Ras-dependent phosphorylation pathways regulate Myc protein stability. Genes Dev..

[77] Morel C., Carlson S.M., White F.M., Davis R.J. (2009). Mcl-1 Integrates the Opposing Actions of Signaling Pathways That Mediate Survival and Apoptosis. Mol. Cell. Biol..

[78] Brunet A., Bonni A., Zigmond M.J., Lin M.Z., Juo P., Hu L.S., Anderson M.J., Arden K.C., Blenis J., Greenberg M.E. (1999). Akt promotes cell survival by phosphorylating and inhibiting a forkhead transcription factor. Cell.

[79] Kops G.J.P.L., Burgering B.M.T. (1999). Forkhead transcription factors: New insights into protein kinase B (c- akt) signaling. J. Mol. Med..

[80] Saxton R.A., Sabatini D.M. (2017). mTOR Signaling in Growth, Metabolism, and Disease. Cell.

[81] Manning B.D., Tee A.R., Logsdon M.N., Blenis J., Cantley L.C. (2002). Identification of the tuberous sclerosis complex-2 tumor suppressor gene product tuberin as a target of the phosphoinositide 3-kinase/Akt pathway. Mol. Cell.

[82] Düvel K., Yecies J.L., Menon S., Raman P., Lipovsky A.I., Souza A.L., Triantafellow E., Ma Q., Gorski R., Cleaver S. (2010). Activation of a metabolic gene regulatory network downstream of mTOR complex 1. Mol. Cell.

[83] Porstmann T., Santos C.R., Griffiths B., Cully M., Wu M., Leevers S., Griffiths J.R., Chung Y.L., Schulze A. (2008). SREBP Activity Is Regulated by mTORC1 and Contributes to Akt-Dependent Cell Growth. Cell Metab..

[84] Inoki K., Li Y., Zhu T., Wu J., Guan K.L. (2002). TSC2 is phosphorylated and inhibited by Akt and suppresses mTOR signalling. Nat. Cell Biol..

[85] Potter C.J., Pedraza L.G., Xu T. (2002). Akt regulates growth by directly phosphorylating Tsc2. Nat. Cell Biol..

[86] Liu P., Gan W., Guo C., Xie A., Gao D., Guo J., Zhang J., Willis N., Su A., Asara J.M. (2015). Akt-mediated phosphorylation of XLF impairs non-homologous end-joining DNA repair. Mol. Cell.

[87] Hu L., Li X., Liu Q., Xu J., Ge H., Wang Z., Wang H., Wang Z., Shi C., Xu X. (2017). UBE2S, a novel substrate of Akt1, associates with Ku70 and regulates DNA repair and glioblastoma multiforme resistance to chemotherapy. Oncogene.

[88] Pham H.T., Nguyen T.T., Nguyen L.P., Han S.S., Lim Y.S., Hwang S.B. (2019). Hepatitis C Virus Downregulates Ubiquitin-Conjugating Enzyme E2S Expression To Prevent Proteasomal Degradation. J. Virol..

[89] Paul A., Wang B. (2017). RNF8- and Ube2S-Dependent Ubiquitin Lysine 11-Linkage Modification in Response to DNA Damage. Mol. Cell.

[90] Altiok S., Batt D., Altiok N., Papautsky A., Downward J., Roberts T.M., Avrahamt H. (1999). Heregulin induces phosphorylation of BRCA1 through phosphatidylinositol 3-kinase/AKT in breast cancer cells. J. Biol. Chem..

[91] Nelson A.C., Lyons T.R., Young C.D., Hansen K.C., Anderson S.M., Holt J.T. (2010). AKT regulates BRCA1 stability in response to hormone signaling. Mol. Cell. Endocrinol..

[92] Kakarougkas A., Jeggo P.A. (2014). DNA DSB repair pathway choice: An orchestrated handover mechanism. Br. J. Radiol..

[93] Brown K.K., Montaser-Kouhsari L., Beck A.H., Toker A. (2015). MERIT40 Is an Akt Substrate that Promotes Resolution of DNA Damage Induced by Chemotherapy. Cell Rep..

[94] Feng L., Huang J., Chen J. (2009). MERIT40 facilitates BRCA1 localization and DNA damage repair. Genes Dev..

[95] Shao G., Patterson-Fortin J., Messick T.E., Feng D., Shanbhag N., Wang Y., Greenberg R.A. (2009). MERIT40 controls BRCA1-Rap80 complex integrity and recruitment to DNA double-strand breaks. Genes Dev..

[96] Jelinic P., Eccles L.A., Tseng J., Cybulska P., Wielgos M., Powell S.N., Levine D.A. (2017). The EMSY threonine 207 phospho-site is required for EMSYdriven suppression of DNA damage repair. Oncotarget.

[97] Hou J., Wang Z., Yang L., Guo X., Yang G. (2014). The function of EMSY in cancer development. Tumor Biol..

[98] Holler M., Grottke A., Mueck K., Manes J., Jücker M., Rodemann H.P., Toulany M. (2016). Dual targeting of Akt and mTORC1 impairs repair of DNA double-strand breaks and increases radiation sensitivity of human tumor cells. PLoS ONE.

[99] Iida M., Harari P.M., Wheeler D.L., Toulany M. (2020). Targeting AKT/PKB to improve treatment outcomes for solid tumors. Mutat. Res. Fundam. Mol. Mech. Mutagen..

[100] Warburg O. (1956). On the origin of cancer cells. Science.

[101] Pavlova N.N., Thompson C.B. (2016). The Emerging Hallmarks of Cancer Metabolism. Cell Metab..

[102] Deberardinis R.J., Chandel N.S. (2016). Fundamentals of cancer metabolism. Sci. Adv..

[103] Srinivas U.S., Tan B.W.Q., Vellayappan B.A., Jeyasekharan A.D. (2019). ROS and the DNA damage response in cancer. Redox Biol..

[104] Kouzarides T. (2007). Chromatin Modifications and Their Function. Cell.

[105] Ui A., Chiba N., Yasui A. (2020). Relationship among DNA double-strand break (DSB), DSB repair, and transcription prevents genome instability and cancer. Cancer Sci..

[106] Dabin J., Fortuny A., Polo S.E. (2016). Epigenome Maintenance in Response to DNA Damage. Mol. Cell.

[107] Lahtz C., Pfeifer G.P. (2011). Epigenetic changes of DNA repair genes in cancer. J. Mol. Cell Biol..

[108] Mcdonald J.T., Kim K., Norris A., Vlashi E., Phillips T.M., Lagadec C., Donna L.D., Ratikan J., Szelag H., Hlatky L. (2010). Ionizing radiation activates the Nrf2 antioxidant response. Cancer Res..

[109] Lu J., Holmgren A. (2014). The thioredoxin antioxidant system. Free Radic. Biol. Med..

[110] Ali S.S., Ahsan H., Zia M.K., Siddiqui T., Khan F.H. (2020). Understanding oxidants and antioxidants: Classical team with new players. J. Food Biochem..

[111] Sies H., de Groot H. (1992). Role of reactive oxygen species in cell toxicity. Toxicol. Lett..

[112] Hlouschek J., Ritter V., Wirsdörfer F., Klein D., Jendrossek V., Matschke J. (2018). Targeting SLC25A10 alleviates improved antioxidant capacity and associated radioresistance of cancer cells induced by chronic-cycling hypoxia. Cancer Lett..

[113] Carracedo A., Cantley L.C., Pandolfi P.P. (2013). Cancer metabolism: Fatty acid oxidation in the limelight. Nat. Rev. Cancer.

[114] Perillo B., Di Donato M., Pezone A., Di Zazzo E., Giovannelli P., Galasso G., Castoria G., Migliaccio A. (2020). ROS in cancer therapy: The bright side of the moon. Exp. Mol. Med..

[115] Sonveaux P. (2017). ROS and radiotherapy: More we care. Oncotarget.

[116] Buj R., Aird K.M. (2018). Deoxyribonucleotide triphosphate metabolism in cancer and metabolic disease. Front. Endocrinol. (Lausanne).

[117] Wang L. (2016). Mitochondrial purine and pyrimidine metabolism and beyond. Nucleosides Nucleotides Nucleic Acids.

[118] Villa E., Ali E.S., Sahu U., Ben-Sahra I. (2019). Cancer cells tune the signaling pathways to empower de novo synthesis of nucleotides. Cancers.

[119] Parker W.B. (2009). Enzymology of purine and pyrimidine antimetabolites used in the treatment of cancer. Chem. Rev..

[120] Polo S.E., Jackson S.P. (2011). POLO, JACKSON—2011—Prot reparo. Genes Dev..

[121] Burgess R.C., Burman B., Kruhlak M., Misteli T. (2014). Activation of DNA damage response signaling by condensed chromatin. Cell Rep..

[122] O’Hagan H.M., Mohammad H.P., Baylin S.B. (2008). Double strand breaks can initiate gene silencing and SIRT1-dependent onset of DNA methylation in an exogenous promoter CpG island. PLoS Genet..

[123] Shanbhan N.M., Rafalska-Metcalf I.U., Balane- Bolivar C., Janicki S.M., Greenberg R.A. (2011). An ATM-Dependent Transcriptional Silencing Program is Transmitted Through Chromatin in Cis to DNA Double Strand Breaks. Cell.

[124] Liu J., Kim J., Oberdoerffer P. (2013). Metabolic modulation of chromatin: Implications for DNA repair and genomic integrity. Front. Genet..

[125] Cuozzo C., Porcellini A., Angrisano T., Morano A., Lee B., Di Pardo A., Messina S., Iuliano R., Fusco A., Santillo M.R. (2007). DNA damage, homology-directed repair, and DNA methylation. PLoS Genet..

[126] Berger S.L., Sassone-Corsi P. (2016). Metabolic signaling to chromatin. Cold Spring Harb. Perspect. Biol..

[127] Uckelmann M., Sixma T.K. (2017). Histone ubiquitination in the DNA damage response. DNA Repair (Amst.).

[128] Meyer H., Weihl C.C. (2014). The VCP/p97 system at a glance: Connecting cellular function to disease pathogenesis. J. Cell Sci..

[129] Schwertman P., Bekker-Jensen S., Mailand N. (2016). Regulation of DNA double-strand break repair by ubiquitin and ubiquitin-like modifiers. Nat. Rev. Mol. Cell Biol..

[130] Bekker-Jensen S., Mailand N. (2010). Assembly and function of DNA double-strand break repair foci in mammalian cells. DNA Repair (Amst.).

[131] Rogakou E.P., Boon C., Redon C., Bonner W.M. (1999). Megabase chromatin domains involved in DNA double-strand breaks in vivo. J. Cell Biol..

[132] Hopfner K.P. (2014). ATP puts the brake on DNA double-strand break repair: A new study shows that ATP switches the Mre11-Rad50-Nbs1 repair factor between signaling and processing of DNA ends Prospects & Overviews K.-P. Hopfner. BioEssays.

[133] Hingorani M.M. (2016). Mismatch binding, ADP-ATP exchange and intramolecular signaling during mismatch repair. DNA Repair.

[134] Locasale J.W., Cantley L.C. (2011). Metabolic flux and the regulation of mammalian cell growth. Cell Metab..

[135] Lunt S.Y., Vander Heiden M.G. (2011). Aerobic Glycolysis: Meeting the Metabolic Requirements of Cell Proliferation. Annu. Rev. Cell Dev. Biol..

[136] Bauer D.E., Hatzivassiliou G., Zhao F., Andreadis C., Thompson C.B. (2005). ATP citrate lyase is an important component of cell growth and transformation. Oncogene.

[137] Haigis M.C., Sinclair D.A. (2010). Mammalian Sirtuins: Biological Insights and Disease Relevance. Annu. Rev. Pathol. Mech. Dis..

[138] Yaku K., Okabe K., Nakagawa T. (2018). NAD metabolism: Implications in aging and longevity. Ageing Res. Rev..

[139] Schreiber V., Dantzer F., Amé J.C., De Murcia G. (2006). Poly(ADP-ribose): Novel functions for an old molecule. Nat. Rev. Mol. Cell Biol..

[140] Beneke S. (2012). Regulation of chromatin structure by poly(ADP-ribosyl)ation. Front. Genet..

[141] Grillo M.A., Colombatto S. (2008). S-adenosylmethionine and its products. Amino Acids.

[142] Yun J., Johnson J.L., Hanigan C.L., Locasale J.W. (2012). Interactions between epigenetics and metabolism in cancers. Front. Oncol..

[143] Cedar H., Bergman Y. (2009). Linking DNA methylation and histone modification: Patterns and paradigms. Nat. Rev. Genet..

[144] Fan C.D., Lum M.A., Xu C., Black J.D., Wang X. (2013). Ubiquitin-dependent regulation of phospho-AKT Dynamics by the ubiquitin E3 LIGASE, NEDD4-1, in the insulin-like growth factor-1 response. J. Biol. Chem..

[145] Maldonado L.Y., Arsene D., Mato J.M., Lu S.C. (2018). Methionine adenosyltransferases in cancers: Mechanisms of dysregulation and implications for therapy. Exp. Biol. Med..

[146] Sun Y., Jiang X., Xu Y., Ayrapetov M.K., Moreau L.A., Whetstine J.R., Price B.D., Cell N., Author B. (2009). Histone H3 methylation links DNA damage detection to activation of the Tip60 tumor suppressor HHS Public Access Author manuscript. Nat. Cell Biol..

[147] Penicud K., Behrens A. (2014). DMAP1 is an essential regulator of ATM activity and function. Oncogene.

[148] Lien E.C., Lyssiotis C.A., Juvekar A., Hu H., Asara J.M., Cantley L.C., Toker A. (2016). Glutathione biosynthesis is a metabolic vulnerability in PI(3)K/Akt-driven breast cancer. Nat. Cell Biol..

[149] Nogueira V., Patra K.C., Hay N. (2018). Selective eradication of cancer displaying hyperactive Akt by exploiting the metabolic consequences of Akt activation. eLife.

[150] Cerutti P., Ghosh R., Oya Y., Amstad P. (1994). The Role of the Cellular Antioxidant Defense in Oxidant Carcinogenesis. Environ. Health Perspect..

[151] Hoxhaj G., Ben-Sahra I., Lockwood S.E., Timson R.C., Byles V., Henning G.T., Gao P., Selfors L.M., Asara J.M., Manning B.D. (2019). Direct stimulation of NADP + synthesis through Akt-mediated phosphorylation of NAD kinase. Science.

[152] Pollak N., Niere M., Ziegler M. (2007). NAD kinase levels control the NADPH concentration in human cells. J. Biol. Chem..

[153] Glorieux C., Calderon P.B. (2017). Catalase, a remarkable enzyme: Targeting the oldest antioxidant enzyme to find a new cancer treatment approach. Biol. Chem..

[154] Senapedis W.T., Kennedy C.J., Boyle P.M., Silver P.A. (2011). Whole genome siRNA cell-based screen links mitochondria to Akt signaling network through uncoupling of electron transport chain. Mol. Biol. Cell.

[155] Weiss C.N., Ito K. (2014). DNA damage response, redox status and hematopoiesis. Blood Cells, Mol. Dis..

[156] Brown G.K. (2000). Glucose transporters: Structure, function and consequences of deficiency. J. Inherit. Metab. Dis..

[157] Petersen M.C., Vatner D.F., Shulman G.I. (2017). Regulation of hepatic glucose metabolism in health and disease. Nat. Rev. Endocrinol..

[158] Fernie A.R., Carrari F., Sweetlove L.J. (2004). Respiratory metabolism: Glycolysis, the TCA cycle and mitochondrial electron transport. Curr. Opin. Plant Biol..

[159] Tozzi M.G., Camici M., Mascia L., Sgarrella F., Ipata P.L. (2006). Pentose phosphates in nucleoside interconversion and catabolism. FEBS J..

[160] Kim J.W., Dang C.V. (2005). Multifaceted roles of glycolytic enzymes. Trends Biochem. Sci..

[161] Fang J., Zhou S.H., Fan J., Yan S.X. (2015). Roles of glucose transporter-1 and the phosphatidylinositol 3-kinase/protein kinase B pathway in cancer radioresistance (Review). Mol. Med. Rep..

[162] Roberts D.J., Tan-Sah V.P., Smith J.M., Miyamoto S. (2013). Akt phosphorylates HK-II at Thr-473 and increases mitochondrial HK-II association to protect cardiomyocytes. J. Biol. Chem..

[163] Roberts D.J., Miyamoto S. (2015). Hexokinase II integrates energy metabolism and cellular protection: Akting on mitochondria and TORCing to autophagy. Cell Death Differ..

[164] Gottlob K., Majewski N., Kennedy S., Kandel E., Robey R.B., Hay N. (2001). Inhibition of early apoptotic events by Akt/PKB is dependent on the first committed step of glycolysis and mitochondrial hexokinase. Genes Dev..

[165] Neary C.L., Pastorino J.G. (2010). Nucleocytoplasmic shuttling of hexokinase II in a cancer cell. Biochem. Biophys. Res. Commun..

[166] Wilson J.E. (2003). Isozymes of mammalian hexokinase: Structure, subcellular localization and metabolic function. J. Exp. Biol..

[167] Novellasdemunt L., Tato I., Navarro-Sabate A., Ruiz-Meana M., Méndez-Lucas A., Perales J.C., Garcia-Dorado D., Ventura F., Bartrons R., Luis Rosa J. (2013). Akt-dependent activation of the heart 6-phosphofructo-2-kinase/fructose-2, 6-bisphosphatase (PFKFB2) isoenzyme by amino acids. J. Biol. Chem..

[168] Liu X., Tan X., Liu P., Wu Y., Qian S., Zhang X. (2018). Phosphoglycerate mutase 1 (PGAM1) promotes pancreatic ductal adenocarcinoma (PDAC) metastasis by acting as a novel downstream target of the PI3K/Akt/mTOR pathway. Oncol. Res..

[169] Qu J., Sun W., Zhong J., Lv H., Zhu M., Xu J., Jin N., Xie Z., Tan M., Lin S.H. (2017). Phosphoglycerate mutase 1 regulates dNTP pool and promotes homologous recombination repair in cancer cells. J. Cell Biol..

[170] Ye G.X., Qin Y., Wang S., Pan D.B., Xu S.Q., Wu C.J., Wang X.M., Wang J., Ye H.L., Shen H.J. (2019). Lamc1 promotes the Warburg effect in hepatocellular carcinoma cells by regulating PKM2 expression through AKT pathway. Cancer Biol. Ther..

[171] Chen C., Liu W.R., Zhang B., Zhang L.M., Li C.G., Liu C., Zhang H., Huo Y.S., Ma Y.C., Tian P.F. (2020). LncRNA H19 downregulation confers erlotinib resistance through upregulation of PKM2 and phosphorylation of AKT in EGFR-mutant lung cancers. Cancer Lett..

[172] Jin L., Alesi G.N., Kang S. (2016). Glutaminolysis as a target for cancer therapy. Oncogene.

[173] Yang L., Venneti S., Nagrath D. (2017). Glutaminolysis: A Hallmark of Cancer Metabolism. Annu. Rev. Biomed. Eng..

[174] Palmada M., Speil A., Jeyaraj S., Böhmer C., Lang F. (2005). The serine/threonine kinases SGK1, 3 and PKB stimulate the amino acid transporter ASCT2. Biochem. Biophys. Res. Commun..

[175] Zhang S., Ren M., Zeng X., He P., Ma X., Qiao S. (2015). Leucine stimulates ASCT2 amino acid transporter expression in porcine jejunal epithelial cell line (IPEC-J2) through PI3K/Akt/mTOR and ERK signaling pathways. Amino Acids.

[176] Wise D.R., Deberardinis R.J., Mancuso A., Sayed N., Zhang X.Y., Pfeiffer H.K., Nissim I., Daikhin E., Yudkoff M., McMahon S.B. (2008). Myc regulates a transcriptional program that stimulates mitochondrial glutaminolysis and leads to glutamine addiction. Proc. Natl. Acad. Sci. USA.

[177] Miller D.M., Thomas S.D., Islam A., Muench D., Sedoris K. (2012). c-Myc and cancer metabolism. Clin. Cancer Res..

[178] Fu Q.F., Liu Y., Fan Y., Hua S.N., Qu H.Y., Dong S.W., Li R.L., Zhao M.Y., Zhen Y., Yu X.L. (2015). Alpha-enolase promotes cell glycolysis, growth, migration, and invasion in non-small cell lung cancer through FAK-mediated PI3K/AKT pathway. J. Hematol. Oncol..

[179] Sun L., Lu T., Tian K., Zhou D., Yuan J., Wang X., Zhu Z., Wan D., Yao Y., Zhu X. (2019). Alpha-enolase promotes gastric cancer cell proliferation and metastasis via regulating AKT signaling pathway. Eur. J. Pharmacol..

[180] Dimri M., Humphries A., Laknaur A., Elattar S., Lee T.J., Sharma A., Kolhe R., Satyanarayana A. (2020). NAD(P)H Quinone Dehydrogenase 1 Ablation Inhibits Activation of the Phosphoinositide 3-Kinase/Akt Serine/Threonine Kinase and Mitogen-Activated Protein Kinase/Extracellular Signal-Regulated Kinase Pathways and Blocks Metabolic Adaptation in Hepatocellular. Hepatology.

[181] Takeo F., Muhammad R.K., Courtney D.D., Johnique T.A.F.J. (2016). The emerging role and targetability of the TCA cycle in cancer metabolism. Discov. Med..

[182] Guo R., Gu J., Zong S., Wu M., Yang M. (2018). Structure and mechanism of mitochondrial electron transport chain. Biomed. J..

[183] Barksdale K.A., Bijur G.N. (2009). The basal flux of Akt in the mitochondria is mediated by heat shock protein-90. J. Neurochem..

[184] Betz C., Stracka D., Prescianotto-Baschong C., Frieden M., Demaurex N., Hall M.N. (2013). MTOR complex 2-Akt signaling at mitochondria-associated endoplasmic reticulum membranes (MAM) regulates mitochondrial physiology. Proc. Natl. Acad. Sci. USA.

[185] Goo C.K., Lim H.Y., Ho Q.S., Too H.P., Clement M.V., Wong K.P. (2012). PTEN/Akt Signaling Controls Mitochondrial Respiratory Capacity through 4E-BP1. PLoS ONE.

[186] McGuire C.M., Forgac M. (2018). Glucose starvation increases V-ATPase assembly and activity in mammalian cells through AMP kinase and phosphatidylinositide 3-kinase/Akt signaling. J. Biol. Chem..

[187] Yu C.C., Yang J.C., Chang Y.C., Chuang J.G., Lin C.W., Wu M.S., Chow L.P. (2013). VCP Phosphorylation-Dependent Interaction Partners Prevent Apoptosis in Helicobacter pylori-Infected Gastric Epithelial Cells. PLoS ONE.

[188] Cerniglia G.J., Dey S., Gallagher-Colombo S.M., Daurio N.A., Tuttle S., Busch T.M., Lin A., Sun R., Esipova T.V., Vinogradov S.A. (2015). The PI3K/Akt pathway regulates oxygen metabolism via pyruvate dehydrogenase (PDH)-E1α phosphorylation. Mol. Cancer Ther..

[189] Deng W., Leu H.B., Chen Y., Chen Y.H., Epperson C.M., Juang C., Wang P.H. (2014). Protein kinase b (pkb/akt1) formed signaling complexes with mitochondrial proteins and prevented glycolytic energy dysfunction in cultured cardiomyocytes during ischemia-reperfusion injury. Endocrinology.

[190] Sutendra G., Kinnaird A., Dromparis P., Paulin R., Stenson T.H., Haromy A., Hashimoto K., Zhang N., Flaim E., Michelakis E.D. (2014). A nuclear pyruvate dehydrogenase complex is important for the generation of Acetyl-CoA and histone acetylation. Cell.

[191] Röhrig F., Schulze A. (2016). The multifaceted roles of fatty acid synthesis in cancer. Nat. Rev. Cancer.

[192] Shimano H., Sato R. (2017). SREBP-regulated lipid metabolism: Convergent physiology-divergent pathophysiology. Nat. Rev. Endocrinol..

[193] Rohlenova K., Neuzil J., Rohlena J. (2016). The role of Her2 and other oncogenes of the PI3K/AKT pathway in mitochondria. Biol. Chem..

[194] Stiles B.L. (2009). PI-3-K and AKT: Onto the mitochondria. Adv. Drug Deliv. Rev..

[195] DeBose-Boyd R.A., Ye J. (2018). SREBPs in Lipid Metabolism, Insulin Signaling, and Beyond. Trends Biochem. Sci..

[196] Cheng C., Geng F., Cheng X., Guo D. (2018). Lipid metabolism reprogramming and its potential targets in cancer. Cancer Commun..

[197] Feng X., Zhang L., Xu S., Shen A. (2020). zong ATP-citrate lyase (ACLY) in lipid metabolism and atherosclerosis: An updated review. Prog. Lipid Res..

[198] Saavedra-García P., Nichols K., Mahmud Z., Fan L.Y.N., Lam E.W.F. (2018). Unravelling the role of fatty acid metabolism in cancer through the FOXO3-FOXM1 axis. Mol. Cell. Endocrinol..

[199] Liu Y., Wang R., Zhang L., Li J., Lou K., Shi B. (2017). The lipid metabolism gene FTO influences breast cancer cell energy metabolism via the PI3K/AKT signaling pathway. Oncol. Lett..

[200] Calvisi D.F., Wang C., Ho C., Ladu S., Lee S.A., Destefanis G., Delogu S., Zimmermann A., Ericsson J., Brozzetti S. (2011). Increased lipogenesis, induced by AKT-mTORC1-RPS6 signaling, promotes development of human hepatocellular carcinoma. Gastroenterology.

[201] Lee J., Ridgway N.D. (2020). Substrate channeling in the glycerol-3-phosphate pathway regulates the synthesis, storage and secretion of glycerolipids. Biochim. Biophys. Acta Mol. Cell Biol. Lipids.

[202] Sivanand S., Rhoades S., Jiang Q., Lee J.V., Benci J., Zhang J., Yuan S., Viney I., Zhao S., Carrer A. (2017). Nuclear Acetyl-CoA Production by ACLY Promotes Homologous Recombination. Mol. Cell.

[203] Luo D.X., Peng X.H., Xiong Y., Liao D.F., Cao D., Li L. (2011). Dual role of insulin-like growth factor-1 in acetyl-CoA carboxylase-alpha activity in human colon cancer cells HCT-8: Downregulating its expression and phosphorylation. Mol. Cell. Biochem..

[204] Hove-Jensen B., Andersen K.R., Kilstrup M., Martinussen J., Switzer R.L., Willemoes M. (2017). Phosphoribosyl Diphosphate (PRPP): Biosynthesis, Enzymology, Utilization, and Metabolic Significanc. Microbiol. Mol. Biol. Rev..

[205] Saha A., Connelly S., Jiang J., Zhuang S., Amador D.T., Phan T., Pilz R.B., Boss G.R. (2014). Akt phosphorylation and regulation of transketolase is a nodal point for amino acid control of purine synthesis. Mol. Cell.

[206] Ben-Sahra I., Hoxhaj G., Ricoult S.J.H., Asara J.M., Manning B.D. (2016). mTORC1 induces purine synthesis through control of the mitochondrial tetrahydrofolate cycle. Science.

[207] Robitaille A.M., Christen S., Shimobayashi M., Cornu M., Fava L.L., Moes S., Prescianotto-Baschong C., Sauer U., Jenoe P., Hall M.N. (2013). Quantitative phosphoproteomics reveal mTORC1 activates de novo pyrimidine synthesis. Science.

[208] Cunningham J.T., Moreno M.V., Lodi A., Ronen S.M., Ruggero D. (2014). Protein and nucleotide biosynthesis are coupled by a single rate-limiting enzyme, PRPS2, to drive cancer. Cell.

[209] Anderson N.M., Mucka P., Kern J.G., Feng H. (2018). The emerging role and targetability of the TCA cycle in cancer metabolism. Protein Cell.

[210] Linder S.J., Mostoslavsky R. (2017). Put Your Mark Where Your Damage Is: Acetyl-CoA Production by ACLY Promotes DNA Repair. Mol. Cell.

[211] Ghobashi A.H., Kamel M.A. (2018). Tip60: Updates. J. Appl. Genet..

[212] Zheng Y., Yang X., Wang C., Zhang S., Wang Z., Li M., Wang Y., Wang X., Yang X. (2020). HDAC6, modulated by miR-206, promotes endometrial cancer progression through the PTEN/AKT/mTOR pathway. Sci. Rep..

[213] Li L., Zheng Y., Zhang W., Hou L., Gao Y. (2020). Scutellarin circumvents chemoresistance, promotes apoptosis, and represses tumor growth by HDAC/miR-34a-mediated down-modulation of Akt/mTOR and NF-κB-orchestrated signaling pathways in multiple myeloma. Int. J. Clin. Exp. Pathol..

[214] Chai R., Fu H., Zheng Z., Liu T., Ji S., Li G. (2017). Resveratrol inhibits proliferation and migration through SIRT1 mediated post-translational modification of PI3K/AKT signaling in hepatocellular carcinoma cells. Mol. Med. Rep..

[215] Dai W., Zhao F., Liu J., Liu H. (2019). ASCT2 Is Involved in SARS-Mediated β-Casein Synthesis of Bovine Mammary Epithelial Cells with Methionine Supply. J. Agric. Food Chem..

[216] Li N., Xue W., Yuan H., Dong B., Ding Y., Liu Y., Jiang M., Kan S., Sun T., Ren J. (2017). AKT-mediated stabilization of histone methyltransferase WHSC1 promotes prostate cancer metastasis. J. Clin. Investig..

[217] Spangle J.M., Dreijerink K.M., Groner A.C., Cheng H., Ohlson C.E., Reyes J., Lin C.Y., Bradner J., Zhao J.J., Roberts T.M. (2016). PI3K/AKT Signaling Regulates H3K4 Methylation in Breast Cancer. Cell Rep..

[218] Deng L., Meng T., Chen L., Wei W., Wang P. (2020). The role of ubiquitination in tumorigenesis and targeted drug discovery. Signal Transduct. Target. Ther..

[219] Zhang R., Liu W., Sun J., Kong Y., Chen C. (2020). Roles of RNF126 and BCA2 E3 ubiquitin ligases in DNA damage repair signaling and targeted cancer therapy. Pharmacol. Res..

[220] Liu B., Chen J., Zhang S. (2019). Emerging role of ubiquitin-specific protease 14 in oncogenesis and development of tumor: Therapeutic implication. Life Sci..

[221] Sharma A., Alswillah T., Singh K., Chatterjee P., Willard B., Venere M., Summers M.K., Almasan A. (2018). USP14 regulates DNA damage repair by targeting RNF168-dependent ubiquitination. Autophagy.

[222] Sheng H., Tang W. (2016). Glycolysis Inhibitors for Anticancer Therapy: A Review of Recent Patents. Recent Pat. Anticancer. Drug Discov..

[223] Jiang X., Sun Q., Li H., Li K., Ren X. (2014). The role of phosphoglycerate mutase 1 in tumor aerobic glycolysis and its potential therapeutic implications. Int. J. Cancer.

[224] Li N., Liu X. (2020). Phosphoglycerate mutase 1: Its glycolytic and non-glycolytic roles in tumor malignant behaviors and potential therapeutic significance. Onco. Targets. Ther..

[225] Stacpoole P.W. (2017). Therapeutic Targeting of the Pyruvate Dehydrogenase Complex/Pyruvate Dehydrogenase Kinase (PDC/PDK) Axis in Cancer. J. Natl. Cancer Inst..

[226] Woolbright B.L., Rajendran G., Harris R.A., Taylor J.A. (2019). Metabolic flexibility in cancer: Targeting the pyruvate dehydrogenase kinase:pyruvate dehydrogenase axis. Mol. Cancer Ther..

[227] Ashton T.M., Gillies McKenna W., Kunz-Schughart L.A., Higgins G.S. (2018). Oxidative phosphorylation as an emerging target in cancer therapy. Clin. Cancer Res..

[228] Sica V., Bravo-San Pedro J.M., Stoll G., Kroemer G. (2020). Oxidative phosphorylation as a potential therapeutic target for cancer therapy. Int. J. Cancer.

[229] Emmings E., Mullany S., Chang Z., Landen C.N., Linder S., Bazzaro M. (2019). Targeting mitochondria for treatment of chemoresistant ovarian cancer. Int. J. Mol. Sci..

[230] Wu Z., Oeck S., West A.P., Mangalhara K.C., Sainz A.G., Newman L.E., Zhang X.O., Wu L., Yan Q., Bosenberg M. (2019). Mitochondrial DNA stress signalling protects the nuclear genome. Nat. Metab..

[231] Liu X., Gong Y. (2019). Isocitrate dehydrogenase inhibitors in acute myeloid leukemia. Biomark. Res..

[232] Fujii T., Khawaja M.R., DiNardo C.D., Atkins J.T., Janku F. (2016). Targeting isocitrate dehydrogenase (IDH) in cancer. Discov. Med..

[233] Guo D., Bell E., Mischel P., Chakravarti A. (2015). Targeting SREBP-1-driven Lipid Metabolism to Treat Cancer. Curr. Pharm. Des..

[234] Zu X.-Y., Zhang Q.-H., Liu J.-H., Cao R.-X., Zhong J., Yi G.-H., Quan Z.-H., Pizzorno G. (2012). ATP Citrate Lyase Inhibitors as Novel Cancer Therapeutic Agents. Recent Pat. Anticancer. Drug Discov..

[235] Granchi C. (2018). ATP citrate lyase (ACLY) inhibitors: An anti-cancer strategy at the crossroads of glucose and lipid metabolism. Eur. J. Med. Chem..

[236] Mohammadi F., Soltani A., Ghahremanloo A., Javid H., Hashemy S.I. (2019). The thioredoxin system and cancer therapy: A review. Cancer Chemother. Pharmacol..

[237] Jia J.J., Geng W.S., Wang Z.Q., Chen L., Zeng X.S. (2019). The role of thioredoxin system in cancer: Strategy for cancer therapy. Cancer Chemother. Pharmacol..

[238] Pramono A.A., Rather G.M., Herman H., Lestari K., Bertino J.R. (2020). NAD- and NADPH-Contributing Enzymes as Therapeutic Targets in Cancer: An Overview. Biomolecules.

[239] Tedeschi P.M., Bansal N., Kerrigan J.E., Abali E.E., Scotto K.W., Bertino J.R. (2016). NAD+ kinase as a therapeutic target in cancer. Clin. Cancer Res..

[240] Tangutoori S., Baldwin P., Sridhar S. (2015). PARP inhibitors: A new era of targeted therapy. Maturitas.

[241] Hou W.H., Chen S.H., Yu X. (2019). Poly-ADP ribosylation in DNA damage response and cancer therapy. Mutat. Res. Rev. Mutat. Res..

[242] Gallyas F., Sumegi B., Szabo C. (2020). Role of akt activation in PARP inhibitor resistance in cancer. Cancers.

[243] Eckschlager T., Plch J., Stiborova M., Hrabeta J. (2017). Histone deacetylase inhibitors as anticancer drugs. Int. J. Mol. Sci..

[244] McClure J.J., Li X., Chou C.J. (2018). Advances and Challenges of HDAC Inhibitors in Cancer Therapeutics.

[245] Li Y., Seto E. (2016). HDACs and HDAC inhibitors in cancer development and therapy. Cold Spring Harb. Perspect. Med..

[246] Zhao L., Duan Y.-T., Lu P., Zhang Z.-J., Zheng X.-K., Wang J.-L., Feng W.-S. (2019). Epigenetic Targets and their Inhibitors in Cancer Therapy. Curr. Top. Med. Chem..

[247] McCabe M.T., Mohammad H.P., Barbash O., Kruger R.G. (2017). Targeting Histone Methylation in Cancer. Cancer J..

[248] Wertz I.E., Murray J.M. (2019). Structurally-defined deubiquitinase inhibitors provide opportunities to investigate disease mechanisms. Drug Discov. Today Technol..

[249] Liu J., Shaik S., Dai X., Wu Q., Zhou X., Wang Z., Wei W. (2015). Targeting the ubiquitin pathway for cancer treatment. Biochim. Biophys. Acta Rev. Cancer.

[250] D’Arcy P., Wang X., Linder S. (2015). Deubiquitinase inhibition as a cancer therapeutic strategy. Pharmacol. Ther..

[251] Liu Z., Xu L. (2018). UBE2S promotes the proliferation and survival of human lung adenocarcinoma cells. BMB Rep..

[252] Huang L., Wang C., Xu H., Peng G. (2020). Targeting citrate as a novel therapeutic strategy in cancer treatment. Biochim. Biophys. Acta Rev. Cancer.

[253] Puigvert J.C., Sanjiv K., Helleday T. (2016). Targeting DNA repair, DNA metabolism and replication stress as anti-cancer strategies. FEBS J..

